# Global burden of disease due to smokeless tobacco consumption in adults: an updated analysis of data from 127 countries

**DOI:** 10.1186/s12916-020-01677-9

**Published:** 2020-08-12

**Authors:** Kamran Siddiqi, Scheherazade Husain, Aishwarya Vidyasagaran, Anne Readshaw, Masuma Pervin Mishu, Aziz Sheikh

**Affiliations:** 1 Department of Health Sciences, University of York, Seebohm Rowntree Building, Heslington, York, YO10 5DD UK; 2 Hull York Medical School, University of York, Heslington, York, YO10 5DD UK; 3 Usher Institute, The University of Edinburgh, Medical School Doorway 3, Teviot Place, Edinburgh, EH8 9AG UK

**Keywords:** Cancer, Chewing, Ischaemic heart disease, Mouth, Oral, Oesophagus, Pharynx, Smokeless tobacco

## Abstract

**Background:**

Smokeless tobacco (ST) is consumed by more than 300 million people worldwide. The distribution, determinants and health risks of ST differ from that of smoking; hence, there is a need to highlight its distinct health impact. We present the latest estimates of the global burden of disease due to ST use.

**Methods:**

The ST-related disease burden was estimated for all countries reporting its use among adults. Using systematic searches, we first identified country-specific prevalence of ST use in men and women. We then revised our previously published disease risk estimates for oral, pharyngeal and oesophageal cancers and cardiovascular diseases by updating our systematic reviews and meta-analyses of observational studies. The updated country-specific prevalence of ST and disease risk estimates, including data up to 2019, allowed us to revise the population attributable fraction (PAF) for ST for each country. Finally, we estimated the disease burden attributable to ST for each country as a proportion of the DALYs lost and deaths reported in the 2017 Global Burden of Disease study.

**Results:**

ST use in adults was reported in 127 countries; the highest rates of consumption were in South and Southeast Asia. The risk estimates for cancers were also highest in this region. In 2017, at least 2.5 million DALYs and 90,791 lives were lost across the globe due to oral, pharyngeal and oesophageal cancers that can be attributed to ST. Based on risk estimates obtained from the INTERHEART study, over 6 million DALYs and 258,006 lives were lost from ischaemic heart disease that can be attributed to ST. Three-quarters of the ST-related disease burden was among men. Geographically, > 85% of the ST-related burden was in South and Southeast Asia, India accounting for 70%, Pakistan for 7% and Bangladesh for 5% DALYs lost.

**Conclusions:**

ST is used across the globe and poses a major public health threat predominantly in South and Southeast Asia. While our disease risk estimates are based on a limited evidence of modest quality, the likely ST-related disease burden is substantial. In high-burden countries, ST use needs to be regulated through comprehensive implementation of the World Health Organization Framework Convention for Tobacco Control.

## Background

Smokeless tobacco (ST) refers to various tobacco-containing products that are consumed by chewing, keeping in the mouth or sniffing, rather than smoking [1]. ST products of many different sorts are used by people in every inhabited continent of the world (Table 1) [1]. For example, in Africa, *toombak* and snuff are commonly used, while in South America, *chimó* is the product of choice. In Australia, indigenous people use *pituri* or *mingkulpa* [2], and in Central Asia, *nasvay* consumption is very common. In North America, plug or snuff are favoured, and even in Western Europe, where ST products are largely banned, there are exemptions allowing people in Nordic countries to use *snus* [3]. All the above products vary in their preparation methods, composition and associated health risks (Table 1), but it is in South and Southeast Asia where the greatest diversity of ST products exists, accompanied by the highest prevalence of use [4]. Here, the level of cultural acceptability is such that ST products are often served like confectionery at weddings and other social occasions.

**Table 1:** Smokeless tobacco products consumed most commonly across the world

Smokeless tobacco products	Regions (WHO)	Countries (highest consumption)	Other ingredients	Preparation and use	pH^**a**^	Nicotine^**a**^ (mg/g)	Total TSNA^**a**^ (ng/g)
Snus (Swedish)	Europe (region A)	Nordic countries (Denmark, Finland, Iceland, Norway, Sweden)	Water, sodium carbonate, sodium chloride, moisturisers, flavouring	A heat treatment process; placed between the gum and upper lip	6.6–7.2	7.8–15.2	601–723
Plug, Snuff (US), Snus (US)	Americas (regions A and B)	The USA, Canada, Mexico	Sweeteners, liquorice	Plug; air cured	4.7–7.8	3.9–40.1	313–76,500
				Dry or moist snuff; finely ground and fire cured			
				Snus; steam cured			
				Snuff; kept between lip and gum, dry snuff can be inhaled too			
Chimó	Americas (region B)	Venezuela, Colombia	Sodium bicarbonate, brown sugar, Mamo’n tree ashes	Tobacco paste made from tobacco leaves; placed between the lip or cheek and gum and left there for some time	6.9–9.4	5.3–30.1	9390
Nass (Naswar)	Europe (region B) and Eastern Mediterranean (region D)	Uzbekistan, Kyrgyzstan, Tajikistan, Afghanistan, Pakistan, Iran	Lime, ash, flavourings (cardamom), indigo	Sundried and powdered; placed between lip or cheek and gum	8.4–9.1	8.9–14.2	478–1380
Toombak	Eastern Mediterranean (region D) and Africa (region D)	Sudan, Chad	Mixed with moist sodium bicarbonate	Fermented and grounded; placed and kept in mouth	7.3–10.1	9.6–28.2	295,000–992,000
Snuff (North and West African)	Africa (region D)	Nigeria, Ghana, Algeria, Cameroon, Chad, Senegal	Dried tobacco leaves mixed with potassium nitrate and other salts	Dry snuff; finely ground and inhaled as a pinch	9.0–9.4	2.5–7.4	1520–2420
				Moist snuff is placed in mouth			
Snuff (South African)	Africa (region E)	South Africa	Dried tobacco leaves mixed with ash	Dry snuff; finely ground and inhaled as a pinch	6.5–10.1	1.2–17.2	1710–20,500
Khaini	South East Asia (regions B and D),Western Pacific (region B), Eastern Mediterranean (region D), and Europe (region A)	India, Bangladesh, Nepal, Bhutan	Slaked lime, menthol, flavourings, areca nut	Shredded; kept in mouth between lips and gum	9.6–9.8	2.5–4.8	21,600–23,900
Zarda		Bangladesh, India, Pakistan, Myanmar, Thailand, Indonesia, Nepal, Maldives, Sri Lanka, UK	Served wrapped in a betel leaf with lime, catechu, areca nuts	Shredded tobacco leaves are boiled with lime and saffron; the mixture is dried then chewed and spat	5.2–6.5	9.5–30.4	5490–53,700
Gutkha		India, Pakistan, Bangladesh, Nepal, Myanmar, Sri Lanka, UK	Betel nut, catechu, flavourings, sweeteners	Commercially manufactured; sucked, chewed, and spat	7.4–8.9	0.2–4.2	83–23,900
Afzal	Eastern Mediterranean (region B)	Oman	Dried tobacco mixed with various additives	Fermented; kept in mouth between lips and gums, users suck the juice, and spit out the rest	10.4	48.7	3573
Iq’mik	Americas (region A)	The USA	Tobacco combined with fungus or plant ash	Involves a burning process to make fungus ash; chewed	11.0	35.0–43.0	15–4910
Rapé	Americas (region B)	Brazil	Tobacco mixed with finely ground plant materials (tonka bean, cinnamon, clove buds, etc.) or alkaline ashes	Nasal snuff; air cured or heated, then pulverised, finely sifted, and mixed	5.2–10.2	6.3–47.6	88–24,200
Pituri/Mingkulpa	Western Pacific (region B)	Australia	Tobacco mixed with wood ash	Chewed as quid, kept in mouth and/or held against skin	5.47–11.6	4.8	15,280

*WHO* World Health Organization, *TSNA* tobacco-specific nitrosamines

^a^Figures are adapted from [1, 2, 18–23]

ST products contain nicotine and are highly addictive. Often, they also contain carcinogens, such as tobacco-specific nitrosamines (TSNA), arsenic, beryllium, cadmium, nickel, chromium, nitrite and nitrate, in varying levels depending on the product [5, 6]. The pH of the products also varies widely, with some (e.g. *khaini*, *zarda*) listing slaked lime among their ingredients [7]. Raising the pH in this way increases the absorption of nicotine and enhances the experience of using the ST product, increasing the likelihood of dependence. The elevated pH also increases the absorption of carcinogens, leading to higher toxicity and greater risk of harm [7].

The harmful nature of many ST products, and the fact that 300 million people around the world use ST [8], make ST consumption a global public health issue. Many ST products lead to different types of head and neck cancers [9, 10]. An increased risk of cardiovascular deaths has been reported [11], and its use in pregnancy is associated with stillbirths and low birth weight [12, 13].

Because of the diversity described above, ST should not be considered as a single product, but rather as groups of products with differences in their toxicity and addictiveness, depending on their composition. As a consequence, it is difficult to estimate the global risks of ST to human health and to agree on international policies for ST prevention and control. Several country-specific studies [14, 15] have been carried out, and in 2015, we published an estimate of the global burden of disease associated with ST use [16]. We used a novel approach, whereby we classified ST products according to their availability in different geographical regions of the world. For example, ST products in South Asia pose a much greater risk to health than those available in Nordic countries, where the manufacturing process removes many of the toxins from the finished product [6, 17]. Using this approach, we estimated the worldwide burden of disease attributable to ST consumption, measured in terms of disability adjusted life years (DALYs) lost and the numbers of deaths in 2010 [16]. Here, we update this estimate to include data up to 2019, providing an indication of how the global ST arena has changed in the intervening years.

## Methods

Our methods for updating the estimates of ST disease burden were broadly the same as those used in our earlier publication; these are well described elsewhere [16]. Here, we will summarise these methods and explain any modification made, particularly in relation to the revised timelines. We assessed disease burden for individual countries by varying their populations’ exposure to ST, using the comparative risk assessment method [15]. These individual estimates were then summarised for 14 World Health Organization (WHO) sub-regions (Additional file 1: Appendix 1) as well as for the world.

We first searched the literature to identify the latest point prevalence of ST use among adults ≥ 15 years in men and women for each country (see Additional file 1: Appendix 2 for detailed methods). We searched for the latest estimates for *x* countries included in our previous study as well as those additional *y* countries where estimates have been made available since 2014 for the first time. We derived single estimates for each country preferring nationally representative surveys using internationally comparable methods over non-standardised national or sub-national surveys.

We also updated risk estimates for individual diseases caused by ST; however, we kept to the original list of conditions, i.e. cancers of the oral cavity, pharynx and oesophagus, ischemic heart disease and stroke. We only searched for papers published since our last literature search; our updated search strategies can be found in Additional file 1: Appendix 3. As before, all searches and data extraction were independently scrutinised by a second researcher and any discrepancies were arbitrated by a third researcher. All case definitions for diseases and exposure (ST use) used in the retrieved articles were checked for accuracy and consistency and all analyses undertaken in these studies were assessed to see if they controlled for key confounders (mainly smoking and alcohol). We assessed study quality using the Newcastle-Ottawa Scale for assessing non-randomised studies in meta-analysis [24]. For all new studies, we log transformed their risk estimates and 95% confidence intervals to effect sizes and standard errors and added these to the rerun of our random-effects meta-analyses to estimate pooled risk estimates for individual conditions. Where possible, we pooled effect sizes to obtain country-specific risk estimates. For all outcomes in the meta-analyses, we conducted a GRADE assessment to assess the quality of evidence. We also pooled these effect sizes to obtain non-specific global risk estimates. Given that the risk varies from country to country, depending upon which products are locally popular, we used country-specific risk estimates where possible. In countries with no estimates, we used estimates of those countries where similar ST products were consumed. For other countries without estimates that consumed ST products known to contain high levels of TSNAs, we applied non-specific global estimates. Where no information was available on the composition of ST, we did not apply any estimates. Details on how these statistically significant estimates were applied to each WHO sub-region can be found in web Additional file 1: Appendix 4.

Based on the extent to which the included studies adjusted for potential confounders, we categorised them as ‘best-adjusted’ and ‘others’. We carried out a sensitivity analysis for all risks and attributable disease burden estimates including only ‘best-adjusted’ studies. A sensitivity analysis was also carried out by estimating risk estimates separating out cohort from case-control studies.

For each country, we used their point prevalence of ST use and the allocated risk estimate for each condition to estimate its population attributable fraction (PAF) as below:

$$ {\displaystyle \begin{array}{c}\mathrm{PAF}={\mathrm{P}}_{\mathrm{e}}\left({\mathrm{RR}}_{\mathrm{e}}-1\right)/\left[1+{\mathrm{P}}_{\mathrm{e}}\left({\mathrm{RR}}_{\mathrm{e}}-1\right)\right]\\ {}{\mathrm{P}}_{\mathrm{e}}=\Pr \mathrm{evalence}\kern0.75em {\mathrm{RR}}_{\mathrm{e}}=\operatorname{Re}\mathrm{lative}\ \mathrm{risk}\end{array}} $$

Using the 2017 Global Burden of Disease (GBD) Study, we also extracted the total disease burden (B) in terms of number of deaths and DALYs lost due to the conditions associated with ST use for both men and women. The attributable burden (AB) due to ST was then estimated in deaths and DALYs lost for these conditions for both men and women using the following equation.

$$ \mathrm{AB}=\mathrm{PAF}\times \mathrm{B} $$

## Results

**Fig. 1:**
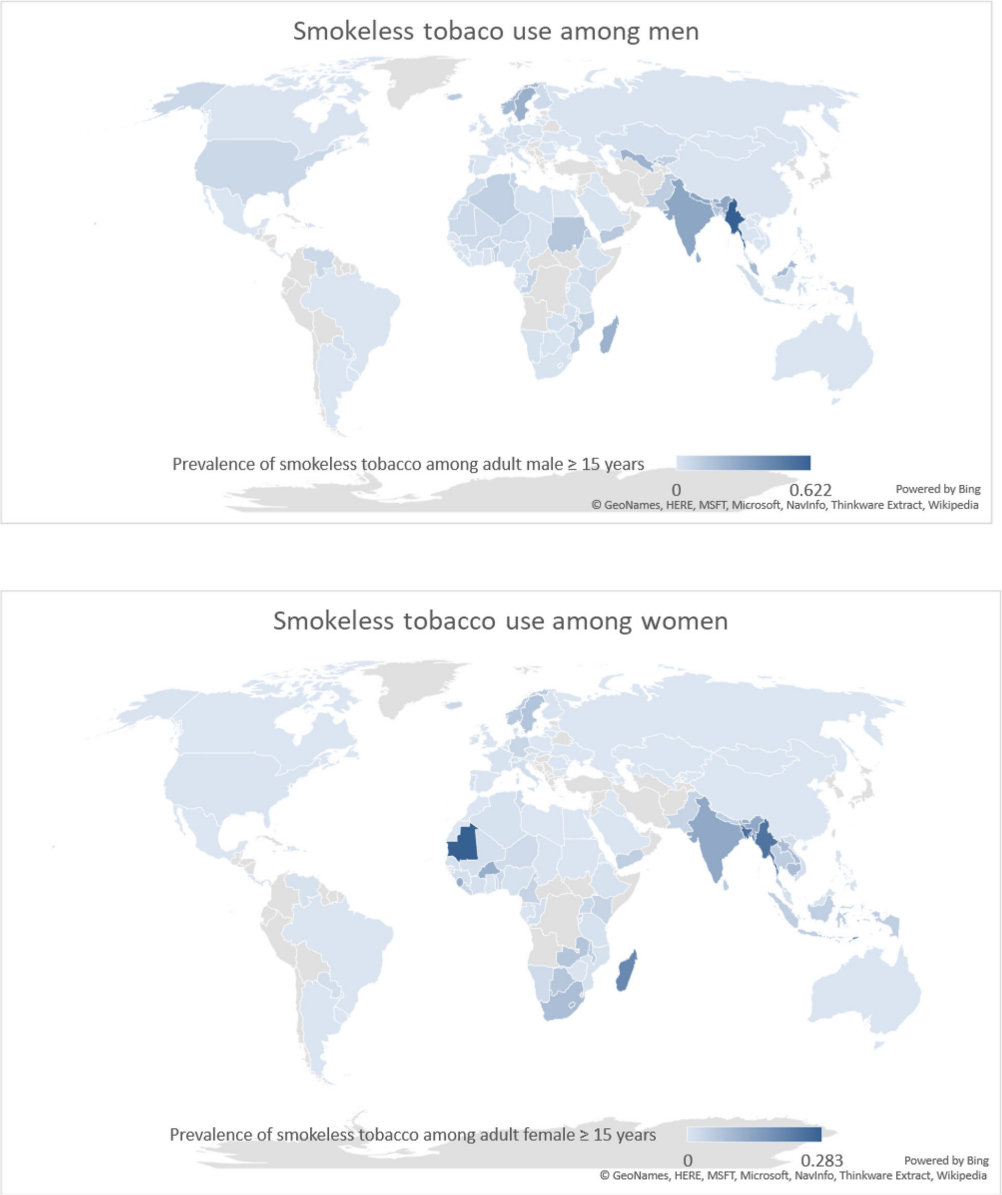
Smokeless tobacco prevalence among men and women

ST consumption was reported in 127 countries (Fig. 1). These estimates were extracted from nationally representative cross-sectional surveys conducted either as part of international (97/127) or national (30/127) health and tobacco surveillance (Additional file 1: Appendix 5a). A variety of age ranges (as young as 15 or as old as 89, including no upper age limit) were used to define adults.

ST consumption was more common among males than females in 95 countries (Table 2). Among males, Myanmar (62.2%), Nepal (31.3%), India (29.6%), Bhutan (26.5%) and Sri Lanka (26.0%) had the highest consumption rates. Among females, Mauritania (28.3%), Timor Leste (26.8%), Bangladesh (24.8%), Myanmar (24.1%) and Madagascar (19.6%) had the highest consumption rates. Within Europe, Sweden (25.0% males, 7.0% females) and Norway (20.1% males, 6.0% females) had the highest ST (snus) consumption rates.

**Table 2:** Prevalence of smokeless tobacco use (%) in different countries of the world according to WHO sub-regional classification

WHO sub-regions	Country	M	F	Source	Year
**Africa (region D)**	Algeria*	10	0.8	Algeria Adult Tobacco Survey [25]	2010
	Benin*	9	3	STEPS [26]	2015
	Burkina Faso*	5.6	11.6	STEPS [26]	2013
	Cameroon*	2.2	3.8	GATS [27]	2013
	Cape Verde	3.5	5.8	STEPS [26]	2007*
	Chad	1.9	0.4	STEPS [26]	2008
	Comoros	7.72	2.99	DHS [28]	2012
	Gabon	0.48	0.34	DHS [28]	2012
	Gambia	0.8	1.4	STEPS [26]	2010 *
	Ghana	1.33	0.2	DHS [28]	2008
	Guinea	1.4	1.5	STEPS [26]	2009
	Liberia*	1.1	3.1	STEPS [26]	2011
	Madagascar	24.66	19.6	DHS [28]	2009
	Mali	5	1.2	STEPS [26]	2007
	Mauritania	5.7	28.3	STEPS [26]	2006
	Niger	4.55	2.3	DHS [29]	2012
	Nigeria*	2.9	0.9	GATS [27]	2012
	Sao Tome & Principe	3.8	1.9	STEPS [26]	2009
	Senegal*	0.3	1	GATS [27]	2015
	Seychelles**	0.3	0.4	The Seychelles Heart Study IV [25]	2013–14
	Sierra Leone	2.9	12.1	STEPS [26]	2009
	Togo	5.1	2.2	STEPS [26]	2010
**Africa (region E)**	*Botswana*	1.5	6.5	STEPS [26]	2014
	*Burundi	0.03	0.31	DHS [28]	2011
	Congo (Brazzaville)	8.3	1.54	DHS [28]	2012
	Congo (Republic)	8.67	3.22	DHS [28]	2013
	Côte d’Ivoire	0.61	1.27	DHS [28]	2012
	Eritrea*	11.6	0.1	STEPS [26]	2011
	Ethiopia*	2.6	0.8	GATS [27]	2016
	Kenya*	5.3	3.8	GATS [27]	2014
	*Lesotho	1.3	9.1	DHS [29]	2009
	*Malawi	1.9	5	STEPS [26]	2009
	Mozambique	10.94	0.82	DHS [28]	2011
	Namibia	1.8	2.3	DHS [29]	2006–07
	Rwanda*	0.6	3.3	STEPS [26]	2012
	*South Africa*	1.4	8.4	South African Social Attitude Survey [25]	2007
	Swaziland*	2.7	1.8	STEPS [26]	2014 *
	*Tanzania	2.03	0.83	DHS [28]	2010
	Uganda*	1.7	3	GATS [27]	2013
	Zambia*	2.2	6.8	STEPS [26]	2017
	Zimbabwe	1.6	0.4	DHS [30]	2011
**Americas (region A)**	*Canada*	0.8	–	CTADS [31]	2015*
	USA	6.5	0.4	ICS [30]	2010
**Americas (region B)**	Argentina	0.1	0.2	GATS [27]	2012
	Barbados	0	0.6	STEPS [26]	2007*
	*Brazil	0.6	0.3	GATS [27]	2008
	Costa Rica**	0.1	0	GATS [27]	2015
	Dominican Republic	1.9	0.3	DHS [29]	2007*
	Grenada	2.2	0.3	STEPS [26]	2011
	Mexico*	0.4	0	GATS [27]	2015
	Panama**	1	0.5	GATS [27]	2013
	Paraguay	3	1.6	STEPS [25]	2011
	St Kitts & Nevis^a^	0.3	0.1	STEPS [26]	2007
	St Lucia**	1.3	0.2	STEPS [26]	2012*
	Trinidad & Tobago	0.5	0.3	STEPS [26]	2011
	*Uruguay**	0.3	–	GATS [27]	2009
	Venezuela	6.2	0.9	National Survey of Drugs in the General Population [25]	2011
**Americas (region D)**	Haiti	–	2.5	DHS [29]	2005–06*
**Eastern Mediterranean (region B)**	Kuwait**	0.5	0	STEPS [26]	2014
	Libya	2.2	0.1	STEPS [26]	2009
	Qatar**	1.3	0	GATS [27]	2013
	Saudi Arabia*	1.5	0.3	Saudi Health Information Survey [25]	2014
	Tunisia	8.6	2.2	ICS [30]	2005–06
**Eastern Mediterranean (region D)**	Egypt*	0.4	0	STEPS [26]	2017
	Iraq*	0.4	0.02	STEPS [26]	2015
	Morocco**	4.4	–	STEPS [26]	2017
	Pakistan*	11.4	3.7	GATS [27]	2014
	Sudan*	14.3	0.2	STEPS [26]	2016
	Yemen	13.7	4.8	National Health and Demographic Survey [25]	2013
**Europe (region A)**	Austria*	2.8	0.5	Representative Survey on Substance Abuse [32]	2015
	Belgium	1.1	0.6	SEBS [33]	2012
	Cyprus	2.1	0.4	SEBS [33]	2012
	Czech Republic*	2.2	1.2	The use of tobacco in the Czech Republic [25]	2015
	Denmark*	2.3	0.9	Monitoring Smoking Habits in the Danish Population [25]	2015
	Finland*	5.6	0.4	Health Behaviour and Health among the Finnish Adult Population [25]	2014
	France	1.2	0.6	SEBS [33]	2012
	Germany	3.4	3.4	SEBS [33]	2012
	Iceland*	13	3	May–December Household Surveys done by Gallup [25]	2015
	Ireland	2.2	0.9	SEBS [33]	2012
	Italy	1.8	1.5	SEBS [33]	2012
	Luxembourg	1.8	1	SEBS [33]	2012
	Malta	5.5	1.5	SEBS [33]	2012
	Netherlands	0.3	0.1	The Dutch Continuous Survey of Smoking Habits [25]	2011
	Norway*	21	6	Statistics Norway Smoking Habits Survey [25]	2015
	Portugal	4.4	1.1	SEBS [33]	2012
	Slovenia	1.8	0.4	SEBS [33]	2012
	Spain	0.4	0.2	SEBS [33]	2012
	Sweden*	25	7	National Survey of Public Health [25]	2015
	Switzerland*	4.2	1.2	Addiction Monitoring survey [25]	2013
	United Kingdom	1.6	0.5	SEBS [33]	2012
**Europe (Region B)**	Azerbaijan*	0.2	0	National study of risk factors for non-communicable diseases [25]	2011
	Armenia	1.8	0	DHS [29]	2005
	Bulgaria	0.3	0	SEBS [33]	2012
	Georgia	1	0.2	Survey of Risk Factors of Non-Communicable Diseases [25]	2010
	*Kazakhstan**	2.8	0	GATS [27]	2014
	Kyrgyzstan*	10.1	0.1	STEPS [26]	2013
	Poland	1	0.1	GATS [27]	2009
	*Romania	0.4	0.2	GATS [27]	2011
	Slovakia*	1.9	0.8	Tobacco and Health Education Survey [25]	2014
	Uzbekistan*	23.2	0.2	STEPS [26]	2014
**Europe (region C)**	Latvia*	0.1	0	Health Behaviour among Latvian Adult Population [25]	2014
	Lithuania	1.2	0.2	SEBS [33]	2012
	Moldova*	0.1	0	DHS [29]	2013
	Russia*	0.8	0.1	GATS [27]	2016
	Ukraine*	0.4	0	GATS [27]	2017
**South East Asia (region B)**	Indonesia*	3.9	4.8	Basic Health Research [25]	2013
	Sri Lanka*	26	5.3	STEPS [26]	2014
	Thailand	1.1	5.2	GATS [27]	2011
**South East Asia (region D)**	Bangladesh*	16.2	24.8	GATS [27]	2017
	Bhutan*	26.5	11	STEPS [26]	2014
	India*	29.6	12.8	GATS [27]	2017
	Maldives*	3.9	1.4	STEPS [26]	2011
	Myanmar*	62.2	24.1	STEPS [26]	2014
	Nepal*	31.3	4.8	STEPS [26]	2013
	Timor Leste*	16.1	26.8	National survey for non-communicable disease risk factors and injuries [34]	2014
**Western Pacific (region A)**	Australia*	0.6	0.3	National Drug Strategy Household Survey [25]	2013
	Brunei Darussalam**	1.3	2.7	Knowledge, Attitudes and Practices Survey on Non-communicable Diseases [25]	2014–15
**Western Pacific (region B)**	Cambodia*	0.8	8.6	National Adult Tobacco Survey of Cambodia [25]	2014
	China	0.7	0	GATS [27]	2010
	Lao People’s Democratic Republic*	0.5	8.6	National Adult Tobacco Survey [25]	2015
	Malaysia*	20.4	0.8	National Health And Morbidity Survey [25]	2015
	Marshall Islands**	13.7	4	STEPS [26]	2002
	Micronesia	22.4	3	STEPS [26]	2002
	Mongolia*	0.8	0.2	STEPS [26]	2015
	Niue**	0.3	0.2	STEPS [26]	2011
	Philippines*	2.7	0.7	GATS [27]	2015
	Vietnam*	0.8	2	GATS [27]	2015

*CTADS* Canadian Tobacco Alcohol and Drugs Survey, *DHS* the Demographic and Health Surveys, *ICS* Individual Country Survey, *GATS* Global Adult Tobacco Survey, *SEBS* The Special Europe Barometer Survey, *STEPS* STEPwise approach to Surveillance, *WHO* World Health Organization

^a^Populations of St Kitts and Nevis are tiny and unlikely to affect our estimates

*Countries included in the earlier paper (*n* = 55), but with updated values

**New countries not included in the earlier paper (*n* = 12)

**Fig. 2:**
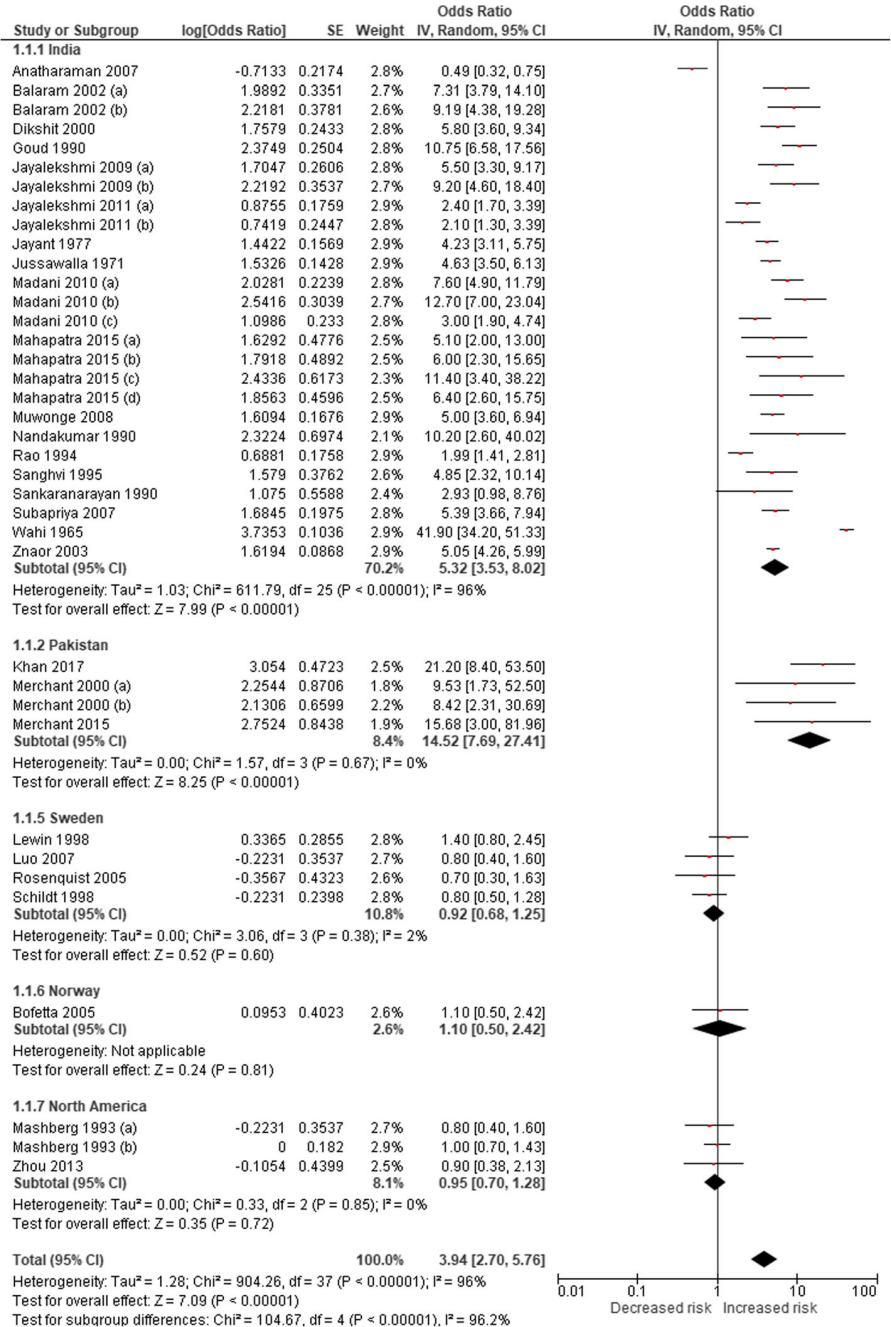
Risk estimates for oral cancers among ever ST users

**Fig. 3:**
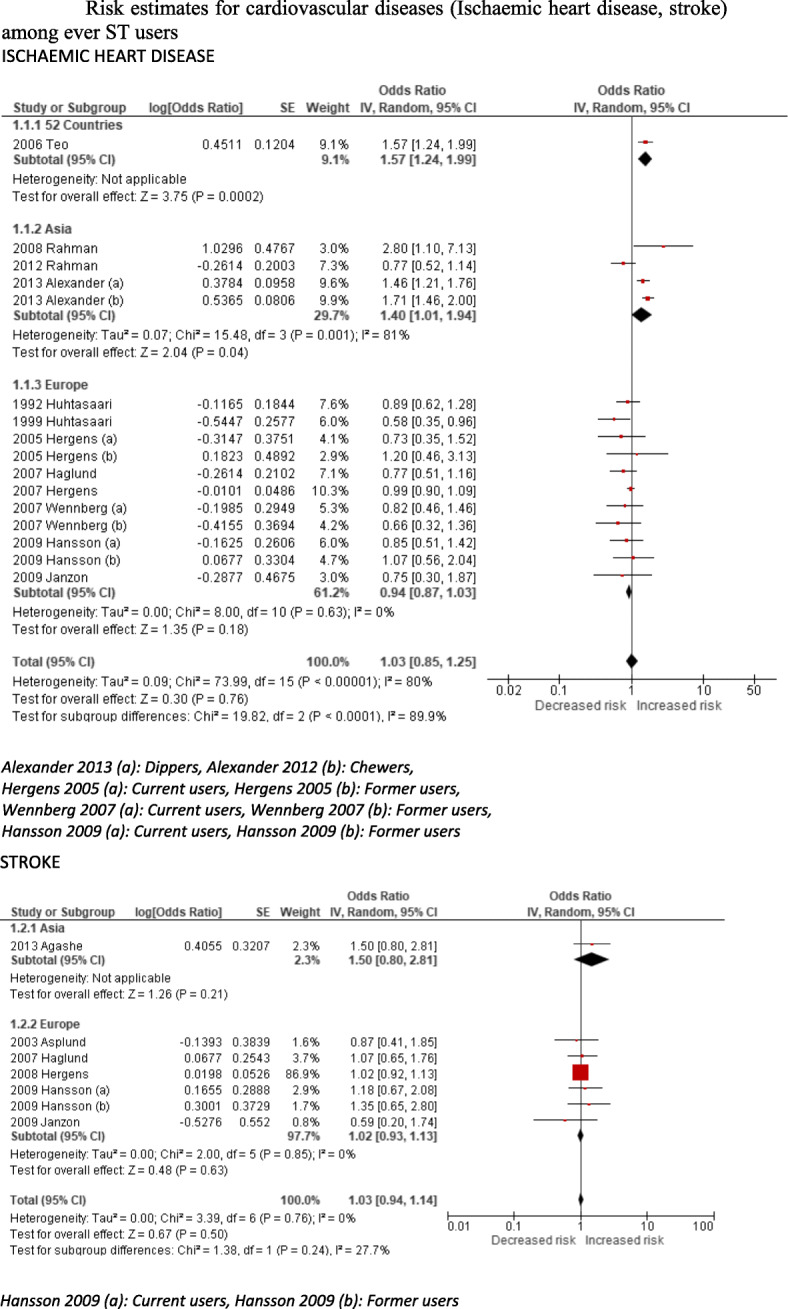
Risk estimates for cardiovascular diseases (ischaemic heart disease, stroke) among ever ST users

Our post-2014 systematic literature search identified an additional four studies demonstrating a causal association between ST and oral cancer; these included two Pakistan-based and one India-based case-control studies and one US-based cohort study (Table 3). No new studies were found for pharyngeal and oesophageal cancers. PRISMA flow diagrams describing the selection process of the studies identified in the literature searches are provided in Additional file 1: Appendix 5b,c. By adding the new studies to the list of studies selected in our first estimates and revising the meta-analyses, we found that the pooled estimates were statistically significant for cancers of the mouth (Fig. 2). The non-specific pooled estimate for oral cancers, based on 36 studies, were 3.94 (95% CI 2.70–5.76). The country-specific relative risk for oral cancers for India was higher (RR 5.32, 95% CI 3.53–8.02) than no-specific estimates and for the USA remained statistically insignificant (RR 0.95, 95% CI 0.70–1.28). Since no new studies were added for pharyngeal and oesophageal cancers, their non-specific risk estimates of 2.23 (95% CI 1.55–3.20) and 2.17 (95% CI 1.70–2.78) remained as per our original estimates, respectively. For cardiovascular diseases, we identified another three Swedish studies for ischaemic heart disease and another two (one in Asia and one in Sweden) for stroke (Table 3). In the absence of any new non-Swedish studies on ischaemic heart disease (Fig. 3), we considered the relative risk (adjusted odds ratio 1.57, 95% CI 1.24–1.99) of myocardial infarction due to ST identified in the 52-country INTERHEART study [35] (conducted across nine WHO regions) as a valid estimate. However, the country-specific (Sweden) relative risk for ischaemic heart disease (RR 0.94, 95% CI 0.87–1.03) and both country-specific (RR 1.02, 95% CI 0.93–1.13 [Sweden]) and non-specific relative risks for stroke (RR 1.03, 95% CI 0.94–1.14) remained statistically insignificant. The GRADE assessment was moderate for oral, pharyngeal and oesophageal cancers and low for IHD (see Additional file 1: Appendix 7).

**Table 3:** Smokeless tobacco use and risk of cancers, ischaemic heart disease, and stroke—studies included in meta-analysis

Country	Study period	Study design	Exposure status	Inclusion of cigarette/alcohol users	Outcome	Odds ratio/relative risk (95% CIs)	Comments	Quality assessment (NOS)^**a**^	Reference
**Cancers**									
India	2001–2004	Case–control	SLT with or without additives	No/no	Oral cancer	0.49 (0.32–0.75)	Exclusive SLT users	Selection**** Comparability** Exposure*	[36]
India	1996–1999	Case–control	Ever SLT users	Yes/yes	Oral cancer	7.31 (3.79–14.1)	Never drinkers adjusted for smoking	Selection**** Comparability** Exposure*	[37]
						9.19 (4.38–19.28)	Never smokers adjusted for alcohol		
India	1982–1992	Case–control	Tobacco quid chewing	Yes/no	Oral cancer	5.80 (3.60–9.34)	Adjusted for smoking	Selection*** Comparability* Exposure*	[38]
					Pharyngeal cancer	1.20 (0.80–1.80)			
					Lung cancer	0.70 (0.40–1.22)			
India	Not clear	Case–control	Chewing tobacco	No/no	Oral cancer	10.75 (6.58–17.56)	Exclusive SLT users	Selection** Comparability* Exposure^0^	[39]
India	1990–1997	Cohort	Current SLT users	No/no	Oral cancer	5.50 (3.30–9.17)	Exclusive SLT users	Selection**** Comparability* Outcome**	[40]
			Former SLT users			9.20 (4.60–18.40)			
India	1990–1997	Cohort	Current SLT user	Yes/yes	Oral cancer	2.40 (1.70–3.39)	Adjusted for smoking and alcohol	Selection**** Comparability* Outcome***	[41]
			Former SLT users			2.10 (1.30–3.39)			
India	Not clear	Case–control	Ever SLT users	No/no	Oral cancer	4.23 (3.11–5.75)	Exclusive SLT users	Selection*** Comparability** Exposure^0^	[42]
					Pharyngeal cancer	2.42 (1.74–3.37)			
					Laryngeal cancer	2.80 (2.07–3.79)			
					Oesophageal cancer	1.55 (1.15–2.07)			
India	1968	Case–control	Tobacco	Yes/no	Oral cancer	4.63 (3.50–6.14)	Exclusive chewers and non-chewers data available	Selection*** Comparability** Exposure^0^	[43]
					Pharyngeal cancer	3.09 (2.31–4.13)			
					Laryngeal cancer	2.29 (1.72–3.05)			
					Oesophageal cancer	3.82 (2.84–5.13)			
India	2005–2006	Case–control	Tobacco flakes	Yes/yes	Oral cancer	7.60 (4.90–11.79)	Adjusted for smoking and alcohol	Selection**** Comparability** Exposure*	[44]
			Gutkha			12.70 (7.00–23.04)			
			Mishiri			3.00 (1.90–4.74)			
India	Not clear	Case–control	Chewing tobacco	Yes/yes	Oral cancer	5.00 (3.60–6.94)	Adjusted for smoking and alcohol	Selection**** Comparability* Exposure*	[45]
India	1982–1984	Case–control	Chewing tobacco	Yes/no	Oral cancer	10.20 (2.60–40.02)	Adjusted for smoking	Selection*** Comparability** Exposure*	[46]
India	1980–1984	Case–control	SLT users	No/no	Oral cancer	1.99 (1.41–2.81)	Exclusive SLT users	Selection** Comparability^0^ Exposure*	[47]
India	1952–1954	Case–control	Chewing tobacco	No/no	Oral cancer	4.85 (2.32–10.14)	Exclusive SLT users	Selection*** Comparability** Exposure^0^	[48]
					Pharyngeal cancer	2.02 (0.94–4.33)			
					Laryngeal cancer	0.76 (0.37–1.56)			
India	1983–1984	Case–control	Snuff (males only)	Yes/yes	Oral cancer	2.93 (0.98–8.76)	Adjusted for smoking and alcohol; adjusted effect size is only among males	Selection*** Comparability^0^ Exposure*	[49]
India	Not given	Case–control	Tobacco chewing	Yes/yes	Oropharyngeal cancer	7.98 (4.11–13.58)^b^	Adjusted for smoking and alcohol	Selection*** Comparability** Exposure^0^	[50]
India	1991–2003	Case–control	Chewing tobacco	No/no	Oral cancer	5.88 (3.66–7.93)	Exclusive SLT users	Selection**** Comparability** Exposure**	[51]
India	1950–1962	Case–control	Tobacco with or without paan or lime	Yes/no	Oral and oropharyngeal cancer	41.90 (34.20–51.33)	Exclusive chewer data available; data of habit was not available for the whole cohort	Selection** Comparability** Exposure^0^	[52]
Pakistan	1996–1998	Case–control	Naswar	Yes/yes	Oral cancer	9.53 (1.73–52.50)	Adjusted for smoking and alcohol	Selection*** Comparability** Exposure*	[53]
			Paan with tobacco			8.42 (2.31–30.69)			
Sweden	1973–2002	Cohort	Snus	Yes/yes	Oral and pharyngeal cancer combined	3.10 (1.50–6.41)	Adjusted for smoking and alcohol	Selection** Comparability** Outcome***	[54]
India	1993–1999	Case–control	Chewing tobacco	Yes/yes	Oral cancer	5.05 (4.26–5.99)	Adjusted for smoking and alcohol	Selection*** Comparability** Exposure*	[55]
					Pharyngeal cancer	1.83 (1.43–2.34)			
					Oesophageal cancer	2.06 (1.62–2.62)			
Norway	1966–2001	Cohort	Chewing tobacco plus oral snuff	No/no	Oral cancer	1.10 (0.50–2.42)	Adjusted for smoking, might be confounded by alcohol use	Selection*** Comparability* Outcome***	[56]
					Oesophageal cancer	1.40 (0.61–3.21)			
					Pancreatic cancer	1.67 (1.12–2.49)			
					Lung cancer	0.80 (0.61–1.05)			
Sweden	1988–1991	Case–control	Oral snuff	Yes/yes	Oral cancer	1.40 (0.80–2.45)	Adjusted for smoking and alcohol	Selection** Comparability** Exposure*	[57]
					Laryngeal cancer	0.90 (0.50–1.62)			
					Oesophageal cancer	1.20 (0.70–2.06)			
					Pharyngeal cancer	0.70 (0.40–1.22)			
Sweden	1969–1992	Cohort	Snus	No/no	Oral cancer	0.80 (0.40–1.60)	Exclusive SLT users	Selection*** Comparability* Outcome***	[58]
					Lung cancer	0.80 (0.50–1.28)			
					Pancreatic cancer	2.00 (1.20–3.33)			
Sweden	2000–2004	Case–control	Oral snuff	Yes/yes	Oral cancer	0.70 (0.30–1.63)	Adjusted for smoking and alcohol	Selection*** Comparability** Exposure**	[59]
Sweden	1980–1989	Case–control	Oral snuff	Yes/yes	Oral cancer	0.80 (0.50–1.28)	Adjusted for smoking and alcohol	Selection** Comparability** Exposure***	[60]
USA	1972–1983	Case–control	Oral snuff	Yes/yes	Oral cancer	0.80 (0.40–1.60)	Not clear if adjusted for smoking and alcohol	Selection** Comparability^0^ Exposure*	[61]
			Chewing tobacco			1.00 (0.70–1.43)			
USA	Not given	Case–control	SLT use	Yes/yes	Oral cancer	0.90 (0.38–2.13)	Adjusted for smoking and alcohol	Selection*** Comparability** Exposure*	[10]
					Pharyngeal cancer	1.59 (0.84–3.01)			
					Laryngeal cancer	0.67 (0.19–2.36)			
India	2001–2004	Case–control	Chewing tobacco	No/no	Pharyngeal cancer	3.18 (1.92–5.27)	Exclusive SLT users	Selection*** Comparability** Exposure*	[62]
					Laryngeal cancer	0.95 (0.52–1.74)			
Pakistan	1998–2002	Case–control	Snuff dipping	No/no	Oesophageal cancer	4.10 (1.30–12.93)	Adjusted for areca nut	Selection*** Comparability** Exposure**	[63]
			Quid with tobacco			14.20 (6.40–31.50)			
India	2008–2012	Case–control	Nass chewing	No/no	Oesophageal cancer	2.88 (2.06–4.03)	Exclusive SLT users	Selection*** Comparability** Exposure**	[64]
			Gutkha chewing			2.87 (0.87–9.47)			
India	2007–2011	Case–control	Oral snuff	Yes/yes	Oesophageal cancer	3.86 (2.46–6.06)	Adjusted for smoking and alcohol	Selection** Comparability** Exposure*	[65]
India	2011–2012	Case–control	Chewing tobacco	Yes/yes	Oesophageal cancer	2.63 (1.53–4.52)	Adjusted for smoking and alcohol	Selection*** Comparability** Exposure*	[66]
Sweden	1995–1997	Case–control	Oral snuff	Yes/yes	Oesophageal adenocarcinoma	1.20 (0.70–2.06)	Adjusted for smoking and alcohol	Selection*** Comparability** Exposure*	[67]
					Squamous cell carcinoma	1.40 (0.90–2.18)			
Sweden	1969–1993	Cohort	Oral snuff	Yes/no	Oesophageal adenocarcinoma	1.30 (0.80–2.11)	Adjusted for smoking	Selection** Comparability* Outcome**	[68]
					Squamous cell carcinoma	1.20 (0.80–1.80)			
Sweden	1974–1985	Cohort	SLT users	No/NA	Lung cancer	0.90 (0.20–4.05)	Adjusted for age, region of origin	Selection*** Comparability* Outcome**	[69]
Morocco	1996–1998	Case–control	SLT users	Yes/no	Lung cancer	1.05 (0.28–3.94)	Adjusted for smoking	Selection** Comparability** Exposure**	[70]
USA	1977–1984	Case–control	SLT users	Yes/no	Oesophageal cancer	1.20 (0.10–14.40)	Adjusted for smoking	Selection*** Comparability** Exposure**	[71]
USA	1986–1989	Case–control	SLT users	Yes/no	Pancreatic cancer	1.40 (0.50–3.92)	Adjusted for smoking	Selection*** Comparability* Exposure**	[72]
USA	2000–2006	Case–control	Chewing tobacco	Yes/yes	Pancreatic cancer	0.60 (0.30–1.20)	Adjusted for smoking and alcohol	Selection**** Comparability** Exposure*	[73]
			Oral snuff			0.50 (0.10–2.50)			
Pakistan	2014–2015	Case–control	Ever use of naswar	Yes/yes	Oral cancer	21.20 (8.40–53.8)	Adjusted for smoking; restricted control for alcohol due to cultural sensitivity	Selection**** Comparability** Exposure***	[74]
India	March–July, 2013	Case–control	Gutkha	Yes/yes	Oral cancer	5.10 (2.00–10.30)	Adjusted for smoking and alcohol	Selection*** Comparability* Exposure**	[75]
			Chewing tobacco			6.00 (2.30–15.70)			
			Supari with tobacco			11.40 (3.40–38.20)			
			Quid with tobacco			6.40 (2.60–15.50)			
Pakistan	1996–1998	Case–control	Quid with tobacco	Yes/yes	Oral cancer	15.68 (3.00–54.90)	Adjusted for smoking and alcohol	Selection** Comparability* Exposure***	[76]
**Cardiovascular diseases (ischaemic heart disease and stroke)**									
52 countries	1999–2003	Case–control	Chewing tobacco	Yes/yes	Myocardial infarction	1.57 (1.24–1.99)	Adjusted for smoking, diet, diabetes, abdominal obesity, exercise, hypertension	Selection**** Comparability** Exposure*	[35]
Pakistan	2005–2011	Case–control	Dippers (Naswar)	No/NA	Myocardial infarction	1.46 (1.21–1.78)	Adjusted for age, gender, region, ethnicity, diet, socioeconomic status	Selection**** Comparability** Exposure**	[77]
			Chewers (Paan/Supari/Gutkha)			1.71 (1.46–2.00)			
Bangladesh	2006–2007	Case–control	Ever SLT users	Yes/NA	Myocardial infarction, angina pectoris	2.80 (1.10–7.30)	Adjusted for age, gender, smoking, hypertension	Selection** Comparability** Exposure**	[78]
Bangladesh	2010	Case–control	Ever SLT users	No/NA	Myocardial infarction, angina pectoris	0.77 (0.52–1.13)	Adjusted for age, gender, area of residence, hypertension, diabetes, stress	Selection*** Comparability** Exposure*	[79]
India	2013	Case–control	Current SLT users	Yes/yes	Stroke	1.50 (0.80–2.79)	Adjusted for age, smoking, alcohol, diabetes, hypertension	Selection** Comparability** Exposure*	[80]
Sweden	1989–1991	Case–control	Current snuff users	No/NA	Myocardial infarction	0.89 (0.62–1.29)	Adjusted for age	Selection**** Comparability** Exposure*	[81]
Sweden	1991–1993	Case–control	Current snuff users	No/NA	Myocardial infarction	0.58 (0.35–0.94)	Adjusted for heredity, education, marital status, hypertension, diabetes, cholesterol	Selection**** Comparability** Exposure**	[82]
Sweden	1985–2000	Case–control	Current snuff users	No/NA	Stroke	0.87 (0.41–1.83)	Adjusted for education, marital status, diabetes, hypertension, cholesterol	Selection**** Comparability** Exposure**	[83]
Sweden	1998–2005	Case–control	Current snuff users	No/NA	Myocardial infarction	0.73 (0.35–1.50)	Adjusted for age, hospital catchment area	Selection*** Comparability** Exposure**	[84]
			Former snuff users			1.20 (0.46–3.10)			
Sweden	1988–2003	Cohort	Current use of snuff	No/NA	Ischaemic heart disease	0.77 (0.51–1.15)	Adjusted for age, socioeconomic status, residential area, self-reported health, longstanding illnesses, physical activity	Selection*** Comparability** Outcome***	[85]
					Stroke	1.07 (0.65–1.77)			
Sweden	1978–2004	Cohort	Ever snuff users	No/NA	Myocardial infarction	0.99 (0.90–1.10)	Adjusted for age, BMI, region of residence	Selection** Comparability** Outcome***	[86]
Sweden	1985–1999	Case–control	Current snuff users	No/NA	Myocardial infarction	0.82 (0.46–1.43)	Adjusted for BMI, leisure time, physical activity, education, cholesterol	Selection**** Comparability** Exposure*	[87]
			Former snuff users			0.66 (0.32–1.34)			
Sweden	1978–2003	Cohort	Ever snuff users	No/NA	Stroke	1.02 (0.92–1.13)	Adjusted for age, BMI, region of residence	Selection** Comparability** Outcome***	[88]
Sweden	1998–2005	Cohort	Current snuff users	No/NA	Ischaemic heart disease	0.85 (0.51–1.42)	Adjusted for age, hypertension, diabetes, cholesterol	Selection*** Comparability** Outcome*	[89]
			Former snuff users			1.07 (0.56–2.04)			
			Current snuff users		Stroke	1.18 (0.67–2.08)			
			Former snuff users			1.35 (0.65–2.82)			
Sweden	1991–2004	Cohort	Current snuff users	No/NA	Myocardial infarction	0.75 (0.30–1.87)	Adjusted for age, marital status, occupation, diabetes, BMI, hypertension, physical activity	Selection*** Comparability** Outcome**	[90]
					Stroke	0.59 (0.20–1.50)			

*BMI* body mass index, *NA* not applicable, *NOS* Newcastle-Ottawa Scale, *SLT* smokeless tobacco

^a^NOS for assessing the quality of non-randomised studies in meta-analyses based on selection, comparability, and exposure/outcome. Number of stars (*) indicates the number of criteria met for each of these three categories

^b^Effect sizes are for oral and pharyngeal cancers combined and were included in the meta-analysis for oral cancer only

We found that most of the included studies adjusted for potential confounders (35/38 for oral, 10/10 for pharyngeal and 15/16 for oesophageal cancers; and 13/16 for IHD) and classified as providing ‘best adjusted’ estimates. According to a sensitivity analysis restricted to only ‘best-adjusted’ studies, the overall risk estimates (RR/OR) for oral cancer increased from 3.94 to 4.46 and for oesophageal cancer from 2.17 to 2.22 (see Additional file 1: sensitivity analysis #1). Separate risk estimates for cohort and case-control studies are included in the Additional file 1: sensitivity analysis #2).

The above risk estimates were included in the mathematical model to estimate the population attributable fraction (PAF), as follows (also see Additional file 1, Appendix 4 for detailed justification): For oral, pharyngeal and oesophageal cancers, Sweden- and US-based country-specific risk estimates were applied to Europe A and America A regions, respectively. Similarly, India-based country-specific risk estimates were applied to Southeast Asia B and D and Western Pacific B regions. No risk estimates were applied to Europe C due to the non-existence of any risk estimates or information about the toxicity of ST products. For all other regions, non-specific country estimates were applied. A few exceptions were made to the above assumptions: a Pakistan-based country-specific estimate was applied for oral cancers for Pakistan and an India-based estimate for the other two cancers; for the UK, India-based country specific estimates were applied due to the predominant use of South Asian products in the country. For ischaemic heart disease, the INTERHEART disease estimates were applied to all WHO regions except two, i.e. Europe A due to the availability of Sweden-based country specific estimates and Europe C due to the non-availability of relevant information. As previously stated, an exception was made for the UK and the INTERHEART estimates were applied.

According to our 2017 estimates, 2,556,810 DALYs lost and 90,791 deaths due to oral, pharyngeal and oesophageal cancers can be attributed to ST use across the globe (Table 4). By applying risk estimates obtained from the INTERHEART study, 6,135,017 DALYs lost and 258,006 deaths from ischaemic heart disease can be attributed to ST use. The overall global disease burden due to ST use amounts to 8,691,827 DALYs lost and 348,798 deaths. The attributable disease burden estimates when restricted to only ‘best adjusted’ studies, did not change significantly; the DALYs lost attributable to ST increased to 8,698,142 and deaths to 349,222.

**Table 4:** Number of deaths and DALYs lost from SLT use in 2017, by WHO sub-region as defined in Additional file 1: Appendix 1

WHO sub-regions^a^	Mouth cancer			Pharyngeal cancer			Oesophageal cancer			Ischaemic heart disease			All causes		
	M	F	All	M	F	All	M	F	All	M	F	All	M	F	All
**Deaths**															
Africa D	184	83	267	120	37	157	294	124	418	3414	1497	4911	4012	1741	5753
Africa E	305	149	454	95	41	136	449	276	725	2231	1797	4027	3079	2263	5343
Americas A	0	0	0	0	0	0	0	0	0	10,298	565	10,863	10,298	565	10,863
Americas B	1189	112	1301	46	4	50	103	12	115	1275	260	1535	2613	389	3001
Americas D	0	3	3	0	1	1	0	2	2	0	76	76	0	82	82
Eastern Mediterranean B	27	3	31	21	1	22	13	1	14	818	122	940	879	128	1007
Eastern Mediterranean D	5488	3756	9244	611	138	749	752	269	1021	13,062	1982	15,045	19,913	6146	26,059
Europe A	69	14	84	30	3	33	246	42	288	0	0	0	346	60	405
Europe B	286	5	291	85	1	86	189	2	192	6552	163	6715	7112	170	7283
Europe C	0	0	0	0	0	0	0	0	0	0	0	0	0	0	0
Southeast Asia B	663	467	1130	394	148	542	260	123	383	5014	3349	8363	6330	4087	10,418
Southeast Asia D	25,966	9829	35,795	16,378	4499	20,876	9366	3493	12,859	147,065	50,509	197,573	198,774	68,329	267,103
Western Pacific A	8	2	11	3	1	4	8	2	10	53	23	76	73	27	100
Western Pacific B	781	173	954	611	44	655	1841	49	1890	7084	798	7883	10,317	1065	11,382
Worldwide	34,966	14,597	49,563	18,394	4918	23,312	13,519	4397	17,916	196,867	61,140	258,006	263,746	85,052	348,798
**DALYs**															
Africa D	5350	2499	7849	3823	1245	5068	7860	3166	11,027	78,500	31,152	109,651	95,533	38,062	133,595
Africa E	9242	4105	13,348	3174	1323	4497	12,358	6590	18,948	59,082	32,930	92,012	83,856	44,948	128,804
Americas A	0	0	0	0	0	0	0	0	0	180,756	6870	187,626	180,756	6870	187,626
Americas B	2283	315	2598	1321	104	1425	2562	261	2823	28,177	4397	32,575	34,344	5077	39,421
Americas D	0	68	68	0	34	34	0	62	62	0	1745	1745	0	1909	1909
Eastern Mediterranean B	758	90	848	593	42	634	301	23	324	16,420	1919	18,339	18,072	2073	20,145
Eastern Mediterranean D	177,353	126,901	304,254	19,303	4655	23,958	20,904	7393	28,298	324,744	46,679	371,423	542,305	185,628	727,933
Europe A	1618	272	1890	686	76	763	4959	682	5641	0	0	0	7263	1030	8293
Europe B	5714	106	5820	2642	30	2672	4871	55	4926	141,562	2177	143,740	154,789	2369	157,158
Europe C	0	0	0	0	0	0	0	0	0	0	0	0	0	0	0
Southeast Asia B	17,730	10,792	28,523	11,164	4319	15,484	6608	2951	9558	122,177	68,896	191,073	157,679	86,958	244,637
Southeast Asia D	767,549	258,275	1,025,824	471,141	131,531	602,672	252,556	87,759	340,314	3,697,819	1,114,976	4,812,796	5,189,065	1,592,540	6,781,606
Western Pacific A	201	48	249	78	15	93	166	24	191	809	233	1042	1255	320	1575
Western Pacific B	20,556	3795	24,351	18,452	1324	19,776	40,948	1055	42,003	157,624	15,371	172,995	237,580	21,545	259,124
Worldwide	1,008,356	407,266	1,415,621	532,378	144,696	677,074	354,093	110,021	464,114	4,807,671	1,327,346	6,135,017	6,702,497	1,989,330	8,691,827

Among these figures, three quarters of the total disease burden was among men. Geographically, > 85% of the disease burden was in South and Southeast Asia, India accounting for 70%, Pakistan for 7% and Bangladesh for 5% DALYs lost due to ST use (Additional file 1: Appendix 6).

## Discussion

ST consumption is now reported in at least two thirds of all countries; however, health risks and the overall disease burden attributable to ST use vary widely depending on the composition, preparation and consumption of these products. Southeast Asian countries share the highest disease burden not only due to the popularity of ST but also due to the carcinogenic properties of ST products. In countries (e.g. Sweden) where ST products are heavily regulated for their composition and the levels of TSNAs, the risk to the population is minimal.

**Fig. 4:**
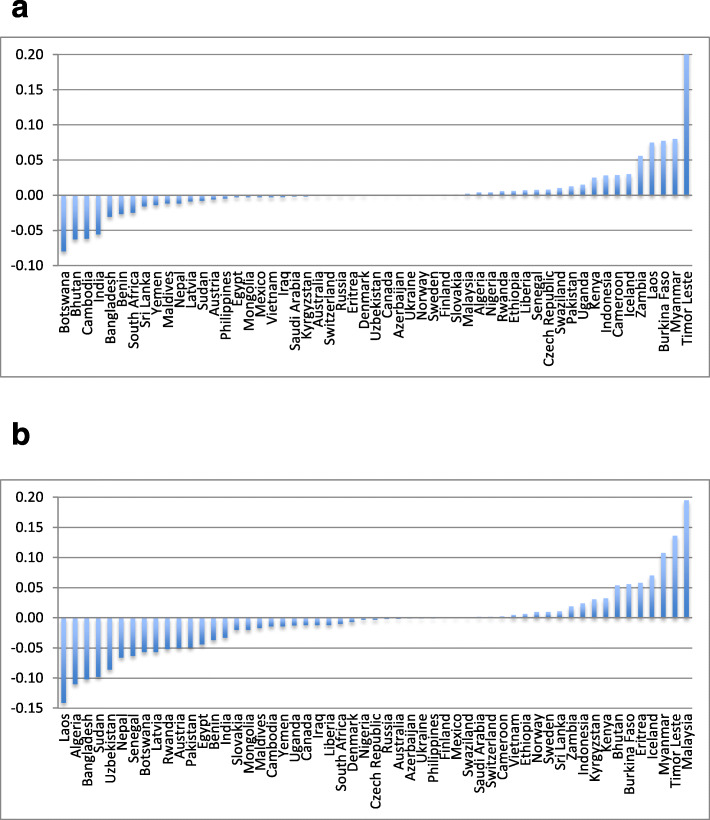
**a** Countries with a proportional change in female ST use between 2015 and 2020 estimates. **b** Countries with a proportional change in male ST use between 2015 and 2020 estimates

We found ST prevalence figures in 12 countries that did not previously report ST use; new figures were also obtained for 55 countries included in the previous estimates [16]. Among these 55 countries: 19 reported a reduction in ST use among both men and women (e.g. Bangladesh, India, Nepal), 14 only among men (e.g. Laos, Pakistan) and eight only among women (e.g. Bhutan, Sri Lanka) (Fig. 4a, b). On the other hand, 13 countries showed an incline in ST use among both men and women (e.g. Indonesia, Myanmar, Malaysia, Timor Leste) and one country (Sweden) among men only. Overall, our updated ST-related disease burden in 2017 was substantially higher than that for 2010—by approximately 50% for cancers and 25% for ischaemic heart disease. This occurred despite a substantial reduction in ST prevalence in India (constituting 70% of the disease burden) and little change in the disease risk estimates. We are now reporting ST use in 12 more countries; however, the main reason for the increased burden of disease was a global rise in the total mortality and DALYs lost—oral, pharyngeal and oesophageal cancers, in particular. The disease burden due to these cancers lags several decades behind the risk exposure. Therefore, a significant reduction in ST-related disease burden as a result of a reduced prevalence will not become apparent for some time to come. Among other studies estimating ST-related global disease burden, our mortality estimates were far more conservative than those reported by Sinha et al. (652,494 deaths); however, their methods were different from ours [9]. Moreover, Sinha et al.’s estimates included a number of additional diseases such as cervical cancer, stomach cancer and stroke. None of these risks were substantiated in our systematic reviews and meta-analyses. On the other hand, our estimates of 2,556,810 DALYs lost and 90,791 deaths due to cancers are close to those estimated by the GBD Study for 2017, i.e.1,890,882 DALYs lost and 75,962 deaths due to cancers [91]. A reason for the slight difference between these two estimates might be that ours included pharyngeal cancers in the estimates while GBD Study only included oral and oesophageal cancers.

Our methods have several limitations. These have been described in detail elsewhere [16] but are summarised here. Our estimates were limited by the availability of reliable data and caveated by several assumptions. The ST use prevalence data were not available for a third of countries despite reports of ST use there. Where prevalence data were available, there were very few studies providing country-specific disease risks—a particular limitation in Africa and South America. In the absence of country-specific risk estimates, the model relied on assuming that countries that share similar ST products also share similar disease risks. For example, oral cancers risk estimates were only available from five countries (India, Norway, Pakistan, Sweden and the USA). For other countries, the extrapolated risks were based on similarities between ST products sold there and in the above five countries. The estimates for ischemic heart disease must be interpreted with caution, in particular, as the risk estimates for most countries were extrapolated from a single (albeit multi-country) study (INTERHEART). However, we excluded those regions from the above extrapolation where the INTERHEART study was not conducted. As previously noted, the total disease burden observed in 2017 is a consequence of risk exposure over several decades. Therefore, the attributable risk based on the prevalence figures gathered in the last few years may not be accurate. If ST prevalence has been declining in a country over the last few decades, the disease burden obtained by applying more recent prevalence figures may underestimate attributable disease burden. This may well be the case in India where ST use has declined by 17% between the 2009 and 2017 GATS surveys [92]. On the other hand, if ST use is on the rise (e.g. in Timor Leste), the attributable disease burden for 2017 could be an overestimate.

While we found a few more recent ST prevalence surveys and observational studies on the risks associated with ST use, big evidence gaps still remain. The ST surveillance data for many countries are either absent or outdated. The biggest gap is in the lack of observational studies on the risks associated with various types of ST used both within and between countries. While longitudinal studies take time, global surveillance of ST products, their chemical composition and risk profile can help improve the precision of future estimates. As cancer registries become more established around the globe, their secondary data analysis can also provide opportunities to estimate ST-related risks.

ST is the main form of tobacco consumption by almost a quarter of all tobacco users in the world. Yet, its regulation and control lags behind that of cigarettes. The diversity in the composition and toxicity of ST products and the role of both formal and informal sectors in its production, distribution and sale make ST regulation a particular challenge. In a recent policy review of 180 countries that are signatories to WHO FCTC, we found that only a handful of countries have addressed ST control at par with cigarettes [93]. The regulatory bar is often much lower for ST than cigarettes [94]. Where ST control policies are present, there are gaps in their enforcement [95]. On the other hand, Sweden has demonstrated what can be achieved through strong regulations; ST-related harm has not only been reduced significantly, but snus is now used to reduce harm from smoking. Countries where ST use is popular and poses risks to health need to prioritise ST control and apply WHO FCTC articles comprehensively and evenly across all forms of tobacco.

## Conclusions

ST is consumed across the globe and poses a major public health threat predominantly in South and Southeast Asia. While our disease risk estimates are based on a limited number of studies with modest quality, the likely disease burden attributable to ST is substantial. In high-burden countries, ST use needs to be regulated through comprehensive implementation and enforcement of the WHO FCTC.

## Supplementary information

**Additional file 1.** Supplementary description of methods and results sections.

## Data Availability

All data generated or analysed during this study are included in this published article and its supplementary information file 1.

## Abbreviations

CI: Confidence intervals
DALYs: Disability-adjusted life years
DHS: Demographic and Health Surveys
GATS: Global Adult Tobacco Survey
ICS: Individual Country Survey
PAF: Population attributable fraction
SEBS: Special Europe Barometer Survey
ST: Smokeless tobacco
STEPS: STEPwise Approach to Surveillance
TSNA: Tobacco-specific nitrosamines
WHO: World Health Organization
