# Supplementary material for: Global burden of disease due to smokeless tobacco consumption in adults: an updated analysis of data from 127 countries
Source: BMC Med. 2020 Aug 12;18:222. doi: 10.1186/s12916-020-01677-9 (PMC7422596; doi:10.1186/s12916-020-01677-9)
Supplement: Supplementary file 1 — Additional file 1. Supplementary description of methods and results sections. [file 12916_2020_1677_MOESM1_ESM.docx]

# Additional file 1: Supplementary description of methods and results sections

## Appendix 1: WHO sub-regional country grouping, by region

| **Africa Region D**: Algeria, Angola, Benin, Burkina Faso, Cameroon, Cape Verde, Chad, Comoros, Equatorial Guinea, Gabon, Gambia, Ghana, Guinea-Bissau, Liberia, Madagascar, Mali, Mauritania, Mauritius, Niger, Nigeria, Sao Tome and Principe, Senegal, Seychelles, Sierra Leone, Togo  **Region E:** Botswana, Burundi, Central African Republic, Congo, Côte d'ivoire, Democratic Republic of Congo, Eritrea, Ethiopia, Kenya, Lesotho, Malawi, Mozambique, Namibia, Rwanda, South Africa, Swaziland, Uganda, Tanzania, Zambia, Zimbabwe  **The Americas Region A:** Canada, USA, Cuba  **Region B:** Antigua and Barbuda, Argentina, Bahamas, Barbados, Belize, Brazil, Chile, Colombia, Costa Rica, Dominica, Dominican Republic, El Salvador, Grenada, Guyana, Honduras, Jamaica, Mexico, Panama, Paraguay, Saint Kitts and Nevis, Saint Lucia, Saint Vincent and the Grenadines, Suriname, Trinidad and Tobago, Uruguay, Venezuela.  **Region D:** Bolivia, Ecuador, Guatemala, Haiti, Nicaragua, Peru  **Eastern Mediterranean Region B:** Bahrain, Iran, Jordan, Kuwait, Lebanon, Libya, Oman, Qatar, Saudi Arabia, Syrian Arab Republic, Tunisia, United Arab Emirates  **Region D:** Afghanistan, Djibouti, Egypt, Iraq, Morocco, Pakistan, Somalia, Sudan, Yemen  **Europe Region A:** Andorra, Austria, Belgium, Croatia, Cyprus, Czech Republic, Denmark, Finland, France, Germany, Greece, Iceland, Ireland, Israel, Italy, Luxembourg, Malta, Monaco, Netherlands, Norway, Portugal, San Marino, Slovenia, Spain, Sweden, Switzerland, UK  **Region B:** Albania, Armenia, Azerbaijan, Bosnia and Herzegovina, Bulgaria, Georgia, Kyrgyzstan, Poland, Romania, Serbia and Montenegro, Slovakia, Tajikistan, Former Yugoslav Republic of Macedonia, Turkey, Turkmenistan, Uzbekistan  **Region C:** Belarus, Estonia, Hungary, Kazakhstan, Latvia, Lithuania, Republic of Moldova, Russian Federation, Ukraine  **Southeast Asia Region: Region B:** Indonesia, Sri Lanka, Thailand  **Region D:** Bangladesh, Bhutan, North Korea, India, Maldives, Myanmar (Burma), Nepal, Timor Leste  **Western Pacific Region: Region A:** Australia, Brunei, Japan, New Zealand, Singapore  **Region B:**Cambodia, China, Cook Islands, Fiji, Kiribati, Laos, Malaysia, Marshall Islands, Micronesia, Mongolia, Nauru, Niue, Palau, Papua New Guinea, Philippines, South Korea, Samoa, Solomon Islands, Tonga, Tuvalu, Vanuatu, Vietnam |
| --- |

Regions are categorized as follows (WHO-approved classifications): A=very low child mortality and very low adult mortality; B=low child mortality and low adult mortality; C=low child mortality and high adult mortality; D=high child mortality and high adult mortality; E=high child mortality and very high adult mortality (Adapted from WHO). ([1](#_heading=h.30j0zll))

## Appendix 2: Search strategy for the prevalence of smokeless tobacco use

For the period 2015–September 2019: SLT prevalence was searched for each country among all adult (≥15 years) populations, and for men and women separately, using the following hierarchy:

- Latest national prevalence data collected as part of an international or regional survey were preferred over an older isolated national or a sub-national survey –
  - We used data from the Global Adult Tobacco Survey (GATS), where available.
  - In its absence, other international (WHO STEPwise approach to Surveillance, The Demographic and Health Surveys), regional (Special Europe Barometer), national, or sub-national surveys were used to extract prevalence data.

Search strategy used in earlier paper (January 1946–February 2015):

*Search terminologies:*

Boolean searching techniques were employed using keywords; ‘smokeless tobacco’, ‘non-burnt tobacco’, ‘spit tobacco’, ‘chewing tobacco’, ‘oral tobacco’, ‘Gutka’, ‘Naswar’, ‘Nas’, ‘Mishri’, ‘Khiwam’, ‘Zarda’, ‘Mawa’, ‘Pan Masala’, ‘Gudhakhu’, ‘Tuibur’, ‘Shamma’, ’Gul’ and ‘Snuff’ etc. combined with names of countries and regions in different search engines described below. (An example of search terminologies and combinations is given below).

1. smokeless tobacco.mp. or tobacco, smokeless/
2. (oral* adj3 tobacco).mp. [mp=title, abstract, original title, name of substance word, subject heading word, keyword heading word, protocol supplementary concept word, rare disease supplementary concept word, unique identifier]
3. (chew* adj3 tobacco).mp. [mp=title, abstract, original title, name of substance word, subject heading word, keyword heading word, protocol supplementary concept word, rare disease supplementary concept word, unique identifier]
4. (spit* adj3 tobacco).mp. [mp=title, abstract, original title, name of substance word, subject heading word, keyword heading word, protocol supplementary concept word, rare disease supplementary concept word, unique identifier]
5. (dip* adj3 tobacco).mp. [mp=title, abstract, original title, name of substance word, subject heading word, keyword heading word, protocol supplementary concept word, rare disease supplementary concept word, unique identifier]
6. gutk?a.mp.
7. kiwam.mp.
8. zarda.mp.
9. mawa.mp.
10. tuibur.mp.
11. shamma.mp.
12. gul.mp.
13. snuf*.mp.
14. snus.mp.
15. chimo.mp.
16. rapé.mp.
17. iq’mik.mp.
18. toombak.mp.
19. tumbaku.mp.
20. mishri.mp.
21. m?sheri.mp. [mp=title, abstract, original title, name of substance word, subject heading word, keyword heading word, protocol supplementary concept word, rare disease supplementary concept word, unique identifier]
22. n?swar.mp. [mp=title, abstract, original title, name of substance word, subject heading word, keyword heading word, protocol supplementary concept word, rare disease supplementary concept word, unique identifier]
23. (p?an adj3 (masala or quid)).mp. [mp=title, abstract, original title, name of substance word, subject heading word, keyword heading word, protocol supplementary concept word, rare disease supplementary concept word, unique identifier]
24. gudak?u.mp. [mp=title, abstract, original title, name of substance word, subject heading word, keyword heading word, protocol supplementary concept word, rare disease supplementary concept word, unique identifier]
25. k?aini.mp. [mp=title, abstract, original title, name of substance word, subject heading word, keyword heading word, protocol supplementary concept word, rare disease supplementary concept word, unique identifier]
26. (maras adj3 (powder or tobacco)).mp. [mp=title, abstract, original title, name of substance word, subject heading word, keyword heading word, protocol supplementary concept word, rare disease supplementary concept word, unique identifier]
27. (quid adj3 (betel or tobacco)).mp. [mp=title, abstract, original title, name of substance word, subject heading word, keyword heading word, protocol supplementary concept word, rare disease supplementary concept word, unique identifier]
28. ((twist or plug) adj3 tobacco).mp. [mp=title, abstract, original title, name of substance word, subject heading word, keyword heading word, protocol supplementary concept word, rare disease supplementary concept word, unique identifier]
29. ((loose leaf or tablet* or toothpaste) adj3 tobacco).mp. [mp=title, abstract, original title, name of substance word, subject heading word, keyword heading word, protocol supplementary concept word, rare disease supplementary concept word, unique identifier]
30. ((pouch* or mix* or powder*) adj3 tobacco).mp. [mp=title, abstract, original title, name of substance word, subject heading word, keyword heading word, protocol supplementary concept word, rare disease supplementary concept word, unique identifier]
31. OR/1-30
32. Epidemiolog$
33. Prevalence$
34. Frequency
35. Survey
36. OR/32-35
37. Region (x) OR Country (x)
38. 31 AND 36 AND 37

*Databases:* Search engines included Medline, Embase, Web of Science, CINAHIL, Pakmed, Informit, Ingenta Connect, Global Health, AJLOL African Journals Online, Airiti Inc, Academic Search, Pubget, OALster, IndMED, LILACS and Cochrane Database were used. Moreover, Google Scholar, Pubmed Database (January 1946–February 2015) and key websites such as (World Health Organisation (WHO), Action on Smoking and Health (ASH) UK, Action on Smoking and Health (ASH) USA, National Institutes of Health (NIH), and Centres for Disease Control and Prevention (CDC) were also searched.

*Language:* No restrictions

*Inclusion criteria:* Prevalence of smokeless tobacco was extracted from only those surveys and studies, which were designed to investigate the prevalence of smokeless tobacco

## Appendix 3: Search strategy for literature review of disease outcomes associated with smokeless tobacco use

*Databases*

MEDLINE, EMBASE, PsycINFO, CINAHL Plus, Web of Science (including Conference Proceedings Citation Index, accessed via Web of Science™ Core Collection), Scopus, Cochrane Library, African Journals Online (AJOL), Latin American and Caribbean Health Sciences Literature (LILACS), WHO Index Medicus of the Eastern Mediterranean Region (IMEMR), WHO Index Medicus of the South-East Asian Region (IMSEAR), PakMediNet, IndMED, ProQuest Dissertations & Theses A&I, EThOS, Open Grey. Reference lists of selected articles were also used to identify relevant studies.

*Main Keywords and Phrases*

The search for this review was conducted by combining an exhaustive list of terms for smokeless tobacco with terms for specific cancers and cardiovascular disease outcomes. The search strategy used for each disease outcome is provided below.

a) Smokeless tobacco and cancer (*MEDLINE via OVID search strategy*) – for the period 2014 to September 3, 2019

1. smokeless tobacco.mp. or tobacco, smokeless/
2. (oral* adj3 tobacco).mp. [mp=title, abstract, original title, name of substance word, subject heading word, keyword heading word, protocol supplementary concept word, rare disease supplementary concept word, unique identifier]
3. (chew* adj3 tobacco).mp. [mp=title, abstract, original title, name of substance word, subject heading word, keyword heading word, protocol supplementary concept word, rare disease supplementary concept word, unique identifier]
4. (spit* adj3 tobacco).mp. [mp=title, abstract, original title, name of substance word, subject heading word, keyword heading word, protocol supplementary concept word, rare disease supplementary concept word, unique identifier]
5. (dip* adj3 tobacco).mp. [mp=title, abstract, original title, name of substance word, subject heading word, keyword heading word, protocol supplementary concept word, rare disease supplementary concept word, unique identifier]
6. gutk?a.mp.
7. kiwam.mp.
8. zarda.mp.
9. mawa.mp.
10. tuibur.mp.
11. shamma.mp.
12. gul.mp.
13. snuf*.mp.
14. snus.mp.
15. chimo.mp.
16. rapé.mp.
17. iq’mik.mp.
18. toombak.mp.
19. tumbaku.mp.
20. mishri.mp.
21. m?sheri.mp. [mp=title, abstract, original title, name of substance word, subject heading word, keyword heading word, protocol supplementary concept word, rare disease supplementary concept word, unique identifier]
22. n?swar.mp. [mp=title, abstract, original title, name of substance word, subject heading word, keyword heading word, protocol supplementary concept word, rare disease supplementary concept word, unique identifier]
23. (p?an adj3 (masala or quid)).mp. [mp=title, abstract, original title, name of substance word, subject heading word, keyword heading word, protocol supplementary concept word, rare disease supplementary concept word, unique identifier]
24. gudak?u.mp. [mp=title, abstract, original title, name of substance word, subject heading word, keyword heading word, protocol supplementary concept word, rare disease supplementary concept word, unique identifier]
25. k?aini.mp. [mp=title, abstract, original title, name of substance word, subject heading word, keyword heading word, protocol supplementary concept word, rare disease supplementary concept word, unique identifier]
26. (maras adj3 (powder or tobacco)).mp. [mp=title, abstract, original title, name of substance word, subject heading word, keyword heading word, protocol supplementary concept word, rare disease supplementary concept word, unique identifier]
27. (quid adj3 (betel or tobacco)).mp. [mp=title, abstract, original title, name of substance word, subject heading word, keyword heading word, protocol supplementary concept word, rare disease supplementary concept word, unique identifier]
28. ((twist or plug) adj3 tobacco).mp. [mp=title, abstract, original title, name of substance word, subject heading word, keyword heading word, protocol supplementary concept word, rare disease supplementary concept word, unique identifier]
29. ((loose leaf or tablet* or toothpaste) adj3 tobacco).mp. [mp=title, abstract, original title, name of substance word, subject heading word, keyword heading word, protocol supplementary concept word, rare disease supplementary concept word, unique identifier]
30. ((pouch* or mix* or powder*) adj3 tobacco).mp. [mp=title, abstract, original title, name of substance word, subject heading word, keyword heading word, protocol supplementary concept word, rare disease supplementary concept word, unique identifier]
31. OR/1 – 30
32. ((oral$ or bucca$ or 'oral cavit$' or (oral adj mucosa$) or (mouth adj mucosa$) or lip or lips or tongue$ or gingiv$ or palat$ or cheek$ or 'intra oral$' or intraoral$ or gum or labial$) adj3 (tumor$ or tumour$ or cancer$ or carcinoma$ or carcinogen$ or neoplas$ or malignan$ or metastas$ or dysplas$ or lesion$ or ulcer$)).mp.
33. 31 AND 32
34. ((esophag$ or oesophag$) adj5 (tumor$ or cancer$ or carcinoma$ or carcinogen$ or neoplas$ or malignan$ or metasta$ or dysplas$ or lesion$ or ulcer$)).tw,ot.
35. 31 AND 34
36. ((pancrea$ or pancreas$) adj5 (tumor$ or cancer$ or carcinoma$ or carcinogen$ or neoplas$ or malignan$ or metasta$ or dysplas$ or lesion$ or ulcer$)).tw,ot.
37. 31 and 36
38. (lung$ adj5 (tumor$ or cancer$ or carcinoma$ or carcinogen$ or neoplas$ or malignan$ or metasta$ or dysplas$ or lesion$ or ulcer$)).tw,ot.
39. 31 and 38

b) Smokeless tobacco and cardiovascular diseases (*MEDLINE via OVID search strategy*) – for the period 1946 to September 3, 2019

1. smokeless tobacco.mp. or tobacco, smokeless/
2. (oral* adj3 tobacco).mp. [mp=title, abstract, original title, name of substance word, subject heading word, keyword heading word, protocol supplementary concept word, rare disease supplementary concept word, unique identifier]
3. (chew* adj3 tobacco).mp. [mp=title, abstract, original title, name of substance word, subject heading word, keyword heading word, protocol supplementary concept word, rare disease supplementary concept word, unique identifier]
4. (spit* adj3 tobacco).mp. [mp=title, abstract, original title, name of substance word, subject heading word, keyword heading word, protocol supplementary concept word, rare disease supplementary concept word, unique identifier]
5. (dip* adj3 tobacco).mp. [mp=title, abstract, original title, name of substance word, subject heading word, keyword heading word, protocol supplementary concept word, rare disease supplementary concept word, unique identifier]
6. gutk?a.mp.
7. kiwam.mp.
8. zarda.mp.
9. mawa.mp.
10. tuibur.mp.
11. shamma.mp.
12. gul.mp.
13. snuf*.mp.
14. snus.mp.
15. chimo.mp.
16. rapé.mp.
17. iq’mik.mp.
18. toombak.mp.
19. tumbaku.mp.
20. mishri.mp.
21. m?sheri.mp. [mp=title, abstract, original title, name of substance word, subject heading word, keyword heading word, protocol supplementary concept word, rare disease supplementary concept word, unique identifier]
22. n?swar.mp. [mp=title, abstract, original title, name of substance word, subject heading word, keyword heading word, protocol supplementary concept word, rare disease supplementary concept word, unique identifier]
23. (p?an adj3 (masala or quid)).mp. [mp=title, abstract, original title, name of substance word, subject heading word, keyword heading word, protocol supplementary concept word, rare disease supplementary concept word, unique identifier]
24. gudak?u.mp. [mp=title, abstract, original title, name of substance word, subject heading word, keyword heading word, protocol supplementary concept word, rare disease supplementary concept word, unique identifier]
25. k?aini.mp. [mp=title, abstract, original title, name of substance word, subject heading word, keyword heading word, protocol supplementary concept word, rare disease supplementary concept word, unique identifier]
26. (maras adj3 (powder or tobacco)).mp. [mp=title, abstract, original title, name of substance word, subject heading word, keyword heading word, protocol supplementary concept word, rare disease supplementary concept word, unique identifier]
27. (quid adj3 (betel or tobacco)).mp. [mp=title, abstract, original title, name of substance word, subject heading word, keyword heading word, protocol supplementary concept word, rare disease supplementary concept word, unique identifier]
28. ((twist or plug) adj3 tobacco).mp. [mp=title, abstract, original title, name of substance word, subject heading word, keyword heading word, protocol supplementary concept word, rare disease supplementary concept word, unique identifier]
29. ((loose leaf or tablet* or toothpaste) adj3 tobacco).mp. [mp=title, abstract, original title, name of substance word, subject heading word, keyword heading word, protocol supplementary concept word, rare disease supplementary concept word, unique identifier]
30. ((pouch* or mix* or powder*) adj3 tobacco).mp. [mp=title, abstract, original title, name of substance word, subject heading word, keyword heading word, protocol supplementary concept word, rare disease supplementary concept word, unique identifier]
31. OR/1 – 30
32. exp Cardiovascular Diseases/ or cardiovascular disease*.mp.
33. exp Myocardial Infarction/ or myocardial infarc*.mp.
34. heart attack*.mp.
35. exp Heart Arrest/ or heart arrest*.mp.
36. exp Coronary Disease/ or exp Coronary Artery Disease/ or coronary disease*.mp.
37. coronary event*.mp.
38. cardio?vascular mortalit*.mp.
39. cardiac mortalit*.mp.
40. cardio?vascular death*.mp.
41. exp Death, Sudden, Cardiac/ or cardiac death*.mp.
42. exp Cerebrovascular Disorders/ or cerebrovascular disorder*.mp.
43. cerebro?vascular accident*.mp.
44. cerebro?vascular event*.mp.
45. cerebro?vascular disease*.mp.
46. exp Stroke/ or stroke*.mp.
47. brain isch?emia.mp. or exp Brain Ischemia/
48. exp Intracranial Hemorrhages/ or exp Cerebral Hemorrhage/ or intracranial h?emorrhag*.mp.
49. OR/32 – 48
50. 31 AND 49

*Language:* No restrictions on language

*Study selection criteria*

- Participants/population - Not restricted by age, gender or country
- Types of study – Cohort studies and case-control studies that reported cancers and cardiovascular disease outcomes in smokeless tobacco users were included. Cross*-*sectional studies, case series and case reports were excluded.
- Exposure – Any use of smokeless tobacco (current and past); If an identified study included users of both smoked and smokeless forms of tobacco, then the presented risk estimate should have been adjusted for smoking.
- Outcome:
  - Cancers – Studies reporting risk estimates for cancers of oral cavity or oropharynx, hypopharynx, nasopharynx, larynx and lip, oesophagus, lungs, and pancreas among smokeless tobacco users were included.
  - Cardiovascular diseases *–* Studies reporting risk estimates for ischaemic heart disease (IHD), and stroke among smokeless tobacco users were included. Studies that only reported ‘intermediate’ cardiovascular outcomes such as blood pressure or lipid levels were excluded from the review.

## Appendix 4: Risk of diseases applied to different WHO regions and the justification (updated)

| Region | Risk of mouth, pharyngeal and oesophageal cancers and ischaemic heart disease | |
| --- | --- | --- |
|  | **Risk estimates (RR/OR applied)** | **Justification** |
| Africa D | For cancers, non-specific global estimates (oral 3.94; pharyngeal 2.2; oesophageal 2.17) and for ischaemic heart disease, INTERHEART study estimates (1.57) | No country specific risk data available from the region but there is reported use of Tambook (Chad) and snuff (Nigeria, Algeria, Ghana); both products have high pH and TSNA. |
| Africa E | For cancers, non-specific global estimates (oral 3.94; pharyngeal 2.2; oesophageal 2.17) and for ischaemic heart disease, INTERHEART study estimates (1.57) | No country specific risk data available from the region but there is reported use of snuff (South Africa, Uganda and Tanzania) products with high pH and TSNA. Besides, Guthka is also used in East Africa (Uganda, Tanzania) among those of South Asian-origin. |
| Americas A | For cancers, country-specific (US) estimates (all cancers 1.0) and for ischaemic heart disease, INTERHEART study estimates (1.57) | Some risk data available from US-based studies. Same SLT products are used both in the US and Canada. |
| Americas B | For cancers, non-specific global estimates (oral 3.94; pharyngeal 2.2; oesophageal 2.17) and for ischaemic heart disease, INTERHEART study estimates (1.57) | No country specific risk data available. Chimó consumption has been reported (Venezuela, Colombia) which has a moderate level of pH and TSNA. Brazil reported use of a SLT product called Rapé but little information is available on this product. |
| Americas D | For cancers, non-specific global estimates (oral 3.94; pharyngeal 2.2; oesophageal 2.17) and for ischaemic heart disease, INTERHEART study estimates (1.57) | Dry snuff is used traditionally in Haiti, which may be linked to oral cancer. |
| Eastern Mediterranean B | For cancers, non-specific global estimates (oral 3.94; pharyngeal 2.2; oesophageal 2.17) and for ischaemic heart disease, INTERHEART study estimates (1.57) | No country specific risk data available. A commonly used SLT product used in this region is Shammah, which (due to its preparation) is likely to be similar to Tambook in its toxic properties. |
| Eastern Mediterranean D | For cancers, country-specific estimates (oral 14.52 [Pakistan] and pharyngeal 2.6 and oesophageal 2.57 [India]) for Pakistan and non-specific global estimates for the rest (oral 3.94; pharyngeal 2.2; oesophageal 2.17). For ischaemic heart disease, INTERHEART study estimates (1.57) | Country specific risk data only available for Pakistan. SLT products used in Pakistan include Nass, Zarda and Guthka, associated with high PH and TSNA. Tambook is used in Sudan also with high pH and TSNA. Shammah is used in Yemen. |
| Europe A | For cancers, country-specific (Sweden) estimates (oral & pharyngeal 1.0; oesophageal 1.23) for Europe A except for the UK where country-specific (India) estimates (oral 5.32; pharyngeal 2.6; oesophageal 2.57) are applied. Please note that the ST products sold in the UK are similar to those in South Asia. For ischaemic heart disease, country-specific (Sweden) estimates (1.0) for Europe A except for the UK where INTERHEART study estimates (1.57) were applied | Considerable country specific risk data available on snus products used in Nordic countries. Little information on the type of SLT products and the associated risks from the rest of Europe except the UK where Naswar, Zarda, Guthka, and Khaini are used**.** |
| Europe B | For cancers, non-specific global estimates (oral 3.94; pharyngeal 2.2; oesophageal 2.17) and for ischaemic heart disease, INTERHEART study estimates (1.57) | No country specific risk data available. However, there is reported and widespread use of Nass in central Asian countries including Uzbekistan, Kyrgyzstan, and Tajikistan – an SLT product with very high pH and TSNA levels. |
| Europe C | No estimates applied | No country specific risk data available. Little information on the products available. |
| South-East Asia B | For cancers, country-specific (India) estimates (oral 5.32; pharyngeal 2.6; oesophageal 2.57) and for ischaemic heart disease, INTERHEART study estimates (1.57) | No country specific risk data available. However, a wide range of SLT products including betel quid use has been reported in the region (Indonesia, Thailand, Sri Lanka) which are similar to those used in India and Bangladesh. |
| South-East Asia D | For cancers, country-specific (India) estimates (oral 5.32; pharyngeal 2.6; oesophageal 2.57) and for ischaemic heart disease, INTERHEART study estimates (1.57) | Considerable country-specific risk data available mainly from India where common SLT products include Khaini, Zarda, and Guthka. Some data also available from Pakistan and Bangladesh where Nass, Guthka, Zarda and betel quid with tobacco are some of the common products. |
| Western Pacific A | For cancers, non-specific global estimates (oral 3.94; pharyngeal 2.2; oesophageal 2.17) and for ischaemic heart disease, INTERHEART study estimates (1.57) | No country specific risk data available. Snuff and chewing tobacco are available in Australia. Included in the INTERHEART study |
| Western Pacific B | For cancers, country specific (India) estimates (oral 5.32; pharyngeal 2.6; oesophageal 2.57) applied  For ischaemic heart disease, INTERHEART study estimates (1.57) | No country specific risk data available. However, reported use of the SLT products (betel quid with tobacco and areca nut) in the region (Laos, Cambodia, Malaysia, Vietnam), which are similar to those used in South East Asia. |

## Appendix 5: Flow diagrams (PRISMA) of the selection process of articles included in the SLT prevalence and the two reviews to assess risk

### 5a. Smokeless tobacco prevalence (1946–February 2015)

For the period 2015–September 2019: countries with updated prevalence since earlier paper = 55, new country prevalence not included in the earlier paper = 12

**Screening**

**Identification**

**Included**

**Eligibility**

Records identified through database searching
(n = 976)

Additional records identified through other sources
(n = 54)

Records after duplicates removed
(n = 1025)

Records screened
(n = 1025)

Records excluded
(n = 926)

Full-text articles assessed for eligibility
(n = 99)

Full-text articles excluded, with reasons
(n = 44)

National but old surveys (n = 7), Population sub-group surveys (n = 7), Findings reported elsewhere (n =15), Sub-national surveys (n = 15)

Studies included in qualitative synthesis
(n = 55)

Studies included in quantitative synthesis*
(n = 55)

*Only one prevalence report was included for one country. Latest national prevalence data collected as part of an international or regional survey was preferred over an older isolated national or a sub-national survey

### 5b. Cancers

**Screening**

**Identification**

**Included**

**Eligibility**

Records identified through database searching
(n = 6661 + 5372)

Additional records identified through other sources
(n = 17 + 1)

Records after duplicates removed
(n = 6227 + 5302)

Records screened
(n = 6227 + 5302)

Records excluded
(n = 6061 + 5277)

Full-text articles assessed for eligibility
(n = 166 + 25)

Full-text articles excluded, with reasons
(n = 84 + 22)

Not exclusive SLT use/did not report adjusted ORs/RRs (n = 43)

Did not meet exposure criteria (n = 21)

Did not meet outcome criteria (n = 20 + 6)

Review article/pooled analyses (n = 15)

Study design (n = 1)

Studies included in qualitative synthesis
(n = 123 + 3)

Studies included in quantitative synthesis (meta-analysis)

(n = 39 + 3)

### 5c. Cardiovascular diseases

**Screening**

**Identification**

**Included**

**Eligibility**

Records identified through database searching
(n = 3443)

Additional records identified through other sources
(n = 0)

Records after duplicates removed
(n = 3411)

Records screened
(n = 3411)

Records excluded
(n = 3383)

Full-text articles assessed for eligibility
(n = 28)

Full-text articles excluded, with reasons
(n = 13)

Pooled analysis (n = 2)

No comparison group of non-tobacco users (n = 2)

Not exclusive use of SLT (n = 3)

Combined outcome reporting (n = 1)

Only CVD deaths reported as outcomes (n = 5)

Studies included in qualitative synthesis
(n = 15)

Studies included in quantitative synthesis (meta-analysis)

(n = 15)

## Appendix 6: Country-specific disease burden attributable to smokeless tobacco use in South and South-East Asia.

|  |  |  | Bangladesh | Bhutan | India | Indonesia | Maldives | Myanmar | Nepal | Pakistan | Sri Lanka | Thailand | Timor Leste |
| --- | --- | --- | --- | --- | --- | --- | --- | --- | --- | --- | --- | --- | --- |
| Mouth cancer | Deaths | Males | 1074 | 11 | 24072 | 238 | 0 | 423 | 381 | 5408 | 351 | 74 | 4 |
|  |  | Females | 1204 | 3 | 8329 | 196 | 0 | 223 | 66 | 3751 | 57 | 215 | 3 |
|  |  | Total | 2278 | 14 | 32401 | 434 | 1 | 646 | 447 | 9159 | 408 | 288 | 7 |
|  | DALYs | Males | 24448 | 309 | 720426 | 7211 | 11 | 11849 | 10409 | 175148 | 8544 | 1976 | 98 |
|  |  | Females | 26813 | 92 | 223989 | 5439 | 7 | 5540 | 1753 | 126738 | 1287 | 4067 | 82 |
|  |  | Total | 51261 | 401 | 944415 | 12649 | 18 | 17388 | 12161 | 301887 | 9830 | 6043 | 180 |
| Pharyngeal cancer | Deaths | Males | 636 | 6 | 15077 | 228 | 0 | 446 | 210 | 566 | 140 | 26 | 3 |
|  |  | Females | 396 | 1 | 3952 | 99 | 0 | 125 | 23 | 136 | 13 | 35 | 2 |
|  |  | Total | 1031 | 7 | 19030 | 327 | 0 | 571 | 233 | 701 | 153 | 61 | 5 |
|  | DALYs | Males | 17681 | 166 | 434050 | 6770 | 2 | 13460 | 5693 | 17904 | 3620 | 774 | 90 |
|  |  | Females | 12343 | 33 | 114683 | 2980 | 0 | 3737 | 688 | 4571 | 349 | 990 | 47 |
|  |  | Total | 30024 | 199 | 548732 | 9751 | 3 | 17196 | 6381 | 22475 | 3969 | 1764 | 137 |
| Oesophageal cancer | Deaths | Males | 544 | 6 | 7442 | 75 | 0 | 1131 | 240 | 637 | 150 | 35 | 2 |
|  |  | Females | 115 | 1 | 2866 | 30 | 0 | 483 | 26 | 255 | 34 | 60 | 2 |
|  |  | Total | 659 | 7 | 10308 | 104 | 0 | 1615 | 265 | 892 | 183 | 95 | 4 |
|  | DALYs | Males | 13486 | 143 | 200229 | 1956 | 3 | 32647 | 5999 | 18031 | 3705 | 947 | 49 |
|  |  | Females | 2597 | 29 | 74187 | 819 | 1 | 10224 | 670 | 6998 | 768 | 1364 | 50 |
|  |  | Total | 16083 | 172 | 274416 | 2775 | 4 | 42871 | 6669 | 25029 | 4473 | 2310 | 100 |
| IHD | Deaths | Males | 6973 | 53 | 132422 | 3023 | 4 | 4370 | 3195 | 8533 | 1826 | 164 | 47 |
|  |  | Females | 6006 | 14 | 42366 | 2550 | 1 | 1834 | 238 | 1592 | 272 | 528 | 49 |
|  |  | Total | 12979 | 67 | 174788 | 5573 | 5 | 6204 | 3433 | 10125 | 2098 | 692 | 97 |
|  | DALYs | Males | 170796 | 1250 | 3347609 | 78952 | 88 | 103987 | 72990 | 218222 | 39599 | 3627 | 1100 |
|  |  | Females | 135295 | 311 | 938294 | 55582 | 11 | 34782 | 5256 | 37377 | 4669 | 8645 | 1027 |
|  |  | Total | 306092 | 1561 | 4285903 | 134534 | 99 | 138769 | 78246 | 255599 | 44268 | 12272 | 2127 |
| All Deaths |  |  | 16947 | 95 | 236528 | 6438 | 6 | 9036 | 4379 | 20876 | 2842 | 1137 | 113 |
| All DALYS |  |  | 403460 | 2331 | 6053466 | 159709 | 123 | 216224 | 103458 | 604990 | 62540 | 22388 | 2543 |

## Appendix 7: GRADE assessments for the key outcomes

### Oral cancer

|  | | | Rating | Adjustment to score |
| --- | --- | --- | --- | --- |
| **Quality assessment** | No. of studies and Starting score | | 38 observational | 2 |
|  | Factors decreasing confidence | Limitation in study design | None serious^1^ | 0 |
|  |  | Inconsistency | Serious^2^ | -1 |
|  |  | Indirectness | None serious | 0 |
|  |  | Imprecision | None serious | 0 |
|  |  | Publication bias | None serious | 0 |
|  | Factors increasing confidence | Large effect | Moderate evidence^3^ | +1 |
|  |  | Dose-response | Weak evidence | 0 |
|  |  | Mitigated bias and confounding | Moderate evidence^4^ | +1 |
|  | Final GRADE score of quality of evidence | | | 3 |
| **Summary** | Quality of evidence | | | Moderate  ⊕⊕⊕ |
| ^1^ Only longitudinal observational studies were included  ^2^ Strong evidence of heterogeneity found  ^3^ Majority of effect sizes were > 2.00/< 0.50 and significant; pooled estimate was > 2.00 and significant  ^4^ Adjustment for main confounders in majority of included studies | | | | |

### Pharyngeal cancer

|  | | | Rating | Adjustment to score |
| --- | --- | --- | --- | --- |
| **Quality assessment** | No. of studies and Starting score | | 10 observational | 2 |
|  | Factors decreasing confidence | Limitation in study design | None serious^1^ | 0 |
|  |  | Inconsistency | Serious^2^ | -1 |
|  |  | Indirectness | None serious | 0 |
|  |  | Imprecision | None serious | 0 |
|  |  | Publication bias | None serious | 0 |
|  | Factors increasing confidence | Large effect | Moderate evidence^3^ | +1 |
|  |  | Dose-response | Weak evidence | 0 |
|  |  | Mitigated bias and confounding | Moderate evidence^4^ | +1 |
|  | Final GRADE score of quality of evidence | | | 3 |
| **Summary** | Quality of evidence | | | Moderate ⊕⊕⊕ |
| ^1^ Only longitudinal observational studies were included  ^2^ Strong evidence of heterogeneity found  ^3^ Majority of effect sizes were > 2.00/< 0.50 and significant; pooled estimate was > 2.00 and significant  ^4^ Adjustment for main confounders in majority of included studies | | | | |

### Oesophageal cancer

|  | | | Rating | Adjustment to score |
| --- | --- | --- | --- | --- |
| **Quality assessment** | No. of studies and Starting score | | 16 observational | 2 |
|  | Factors decreasing confidence | Limitation in study design | None serious^1^ | 0 |
|  |  | Inconsistency | Serious^2^ | -1 |
|  |  | Indirectness | None serious | 0 |
|  |  | Imprecision | None serious | 0 |
|  |  | Publication bias | None serious | 0 |
|  | Factors increasing confidence | Large effect | Moderate evidence^3^ | +1 |
|  |  | Dose-response | Weak evidence | 0 |
|  |  | Mitigated bias and confounding | Moderate evidence^4^ | +1 |
|  | Final GRADE score of quality of evidence | | | 3 |
| **Summary** | Quality of evidence | | | Moderate  ⊕⊕⊕ |
| ^1^ Only longitudinal observational studies were included  ^2^ Strong evidence of heterogeneity found  ^3^ Half the included effect sizes were > 2.00/< 0.50 and significant; pooled estimate was > 2.00 and significant  ^4^ Adjustment for main confounders in majority of studies | | | | |

### IHD

|  | | | Rating | Adjustment to score |
| --- | --- | --- | --- | --- |
| **Quality assessment** | No. of studies and Starting score | | 16 observational | 2 |
|  | Factors decreasing confidence | Limitation in study design | None serious^1^ | 0 |
|  |  | Inconsistency | Serious^2^ | -1 |
|  |  | Indirectness | None serious | 0 |
|  |  | Imprecision | None serious | 0 |
|  |  | Publication bias | None serious | 0 |
|  | Factors increasing confidence | Large effect | Weak evidence^3^ | 0 |
|  |  | Dose-response | Weak evidence | 0 |
|  |  | Mitigated bias and confounding | Moderate evidence^4^ | +1 |
|  | Final GRADE score of quality of evidence | | | 2 |
| **Summary** | Quality of evidence | | | Low  ⊕⊕ |
| ^1^ Only longitudinal observational studies were included  ^2^ Strong evidence of heterogeneity found  ^3^ Few effect sizes were > 2.00/< 0.50 and significant  ^4^ Adjustment for main confounders in majority of studies | | | | |

## Sensitivity analysis #1 – by best adjusted estimates

| **Outcome** | **All estimates** | | **Best adjusted** | | **Others** | |
| --- | --- | --- | --- | --- | --- | --- |
|  | **No.** | **OR/RR**  **(95% CI)** | **No.** | **OR/RR**  **(95% CI)** | **No.** | **OR/RR**  **(95% CI)** |
| Oral cancer | 38 | 3.94  (2.70 – 5.76) | 35 | 4.46  (3.04 – 6.54) | 3 | 0.97  (0.73 – 1.31) |
| India | 26 | 5.32  (3.53 – 8.02) | 26 | 5.32  (3.53 – 8.02) | 0 | Not estimable |
| Pakistan | 4 | 14.52  (7.69 – 27.41) | 4 | 14.52  (7.69 – 27.41) | 0 | Not estimable |
| Sweden | 4 | 0.92  (0.68 – 1.25) | 4 | 0.92  (0.68 – 1.25) | 0 | Not estimable |
| Norway | 1 | 1.10  (0.50 – 2.42) | 0 | Not estimable | 1 | 1.10  (0.50 – 2.42) |
| USA | 3 | 0.95  (0.70 – 1.28) | 1 | 0.90  (0.38 – 2.13) | 2 | 0.95  (0.69 – 1.31) |
| Pharyngeal cancer | 10 | 2.23  (1.55 – 3.20) | 10 | 2.23  (1.55 – 3.20) | 0 | Not estimable |
| India | 7 | 2.60  (1.76 – 3.85) |  |  |  |  |
| Sweden | 2 | 1.45  (0.34 – 6.21) |  |  |  |  |
| USA | 1 | 1.59  (0.84 – 3.01) |  |  |  |  |
| **Oesophageal cancer** | 16 | 2.17  (1.70 – 2.78) | 15 | 2.22  (1.72 – 2.86) | 1 | 1.40  (0.61 – 3.21) |
| India | 7 | 2.57  (2.20 – 3.00) | 7 | 2.57  (2.20 – 3.00) | 0 | Not estimable |
| Pakistan | 2 | 8.20  (2.45 – 27.47) | 2 | 8.20  (2.45 – 27.47) | 0 | Not estimable |
| Sweden | 5 | 1.26  (1.02 – 1.56) | 5 | 1.26  (1.02 – 1.56) | 0 | Not estimable |
| Norway | 1 | 1.40  (0.61 – 3.21) | 0 | Not estimable | 1 | 1.40  (0.61 – 3.21) |
| USA | 1 | 1.20  (0.10 – 14.40) | 1 | 1.20  (0.10 – 14.40) | 0 | Not estimable |
| IHD | 16 | 1.03  (0.85 – 1.25) | 13 | 1.05  (0.85 – 1.30) | 3 | 0.89  (0.65 – 1.21) |
| Bangladesh | 2 | 1.37  (0.39 – 4.80) | 2 | 1.37  (0.39 – 4.80) | 0 | Not estimable |
| Pakistan | 2 | 1.59  (1.37 – 1.86) | 2 | 1.59  (1.37 – 1.86) | 0 | Not estimable |
| Sweden | 11 | 0.94  (0.87 – 1.03) | 8 | 0.93  (0.84 – 1.03) | 3 | 0.89  (0.65 – 1.21) |
| 52 countries | 1 | 1.57  (1.24 – 1.99) | 1 | 1.57  (1.24 – 1.99) | 0 | Not estimable |

## Sensitivity analysis #2 – by study design

| **Outcome** | **Combined estimate** | | **Cohort studies** | | **Case-control studies** | |
| --- | --- | --- | --- | --- | --- | --- |
|  | **No.** | **OR/RR**  **(95% CI)** | **No.** | **RR**  **(95% CI)** | **No.** | **OR**  **(95% CI)** |
| Oral cancer | 38 | 3.94  (2.70 – 5.76) | 6 | 2.51  (1.36 – 4.62) | 32 | 4.32  (2.82 – 6.62) |
| India | 26 | 5.32  (3.53 – 8.02) | 4 | 3.83  (2.08 – 7.05) | 22 | 5.64  (3.56 – 8.92) |
| Pakistan | 4 | 14.52  (7.69 – 27.41) | 0 | Not estimable | 4 | 14.52  (7.69 – 27.41) |
| Sweden | 4 | 0.92  (0.68 – 1.25) | 1 | 0.80  (0.40 – 1.60) | 3 | 0.95  (0.63 – 1.44) |
| Norway | 1 | 1.10  (0.50 – 2.42) | 1 | 1.10  (0.50 – 2.42) | 0 | Not estimable |
| USA | 3 | 0.95  (0.70 – 1.28) | 0 | Not estimable | 3 | 0.95  (0.70 – 1.28) |
| Pharyngeal cancer | 10 | 2.23  (1.55 – 3.20) | 1 | 3.10  (1.50 – 6.41) | 9 | 2.16  (1.47 – 3.18) |
| India | 7 | 2.60  (1.76 – 3.85) | 0 | Not estimable | 7 | 2.60  (1.76 – 3.85) |
| Sweden | 2 | 1.45  (0.34 – 6.21) | 1 | 3.10  (1.50 – 6.41) | 1 | 0.70  (0.40 – 1.22) |
| USA | 1 | 1.59  (0.84 – 3.01) | 0 | Not estimable | 1 | 1.59  (0.84 – 3.01) |
| **Oesophageal cancer** | 16 | 2.17  (1.70 – 2.78) | 3 | 1.26  (0.94 – 1.68) | 13 | 2.46  (1.89 – 3.19) |
| India | 7 | 2.57  (2.20 – 3.00) | 0 | Not estimable | 7 | 2.57  (2.20 – 3.00) |
| Pakistan | 2 | 8.20  (2.45 – 27.47) | 0 | Not estimable | 2 | 8.20  (2.45 – 27.47) |
| Sweden | 5 | 1.26  (1.02 – 1.56) | 2 | 1.24  (0.91 – 1.69) | 3 | 1.28  (0.96 – 1.71) |
| Norway | 1 | 1.40  (0.61 – 3.21) | 1 | 1.40  (0.61 – 3.21) | 0 | Not estimable |
| USA | 1 | 1.20  (0.10 – 14.40) | 0 | Not estimable | 1 | 1.20  (0.10 – 14.40) |
| IHD | 16 | 1.03  (0.85 – 1.25) | 5 | 0.97  (0.89 – 1.06) | 11 | 1.09  (0.85 – 1.39) |
| Bangladesh | 2 | 1.37  (0.39 – 4.80) | 0 | Not estimable | 2 | 1.37  (0.39 – 4.80) |
| Pakistan | 2 | 1.59  (1.37 – 1.86) | 0 | Not estimable | 2 | 1.59  (1.37 – 1.86) |
| Sweden | 11 | 0.94  (0.87 – 1.03) | 5 | 0.97  (0.89 – 1.06) | 6 | 0.78  (0.62 – 0.98) |
| 52 countries | 1 | 1.57  (1.24 – 1.99) | 0 | Not estimable | 1 | 1.57  (1.24 – 1.99) |

## References of appendix:

1. Mutangadura GB. World Health Report 2002: Reducing Risks, Promoting Healthy Life: World Health Organization, Geneva, 2002, 250 pages, US $13.50, ISBN 9-2415-6207-2. No longer published by Elsevier; 2004.
